# Connexin-43 hemichannels orchestrate NOD-like receptor protein-3 (NLRP3) inflammasome activation and sterile inflammation in tubular injury

**DOI:** 10.1186/s12964-023-01245-7

**Published:** 2023-09-28

**Authors:** Elena Roger, Christos E. Chadjichristos, Panagiotis Kavvadas, Gareth W. Price, Chelsy L. Cliff, Safia Hadjadj, Jessy Renciot, Paul E. Squires, Claire E. Hills

**Affiliations:** 1 Batiment Recherche, INSERM, UMR-S1155, Tenon Hospital, 4 Rue de la Chine, Paris, 75020 France; 2 Faculty of Medicine, Sorbonne University, Paris, 75013 France; 3 Joseph Banks Laboratories, School of Life and Environmental Sciences, University of Lincoln, Lincoln, LN6 7DL UK

**Keywords:** Connexins, Connexin 43, Hemichannels, Inflammation, Inflammasomes, Kidney disease, Tubulointerstitial inflammation, Peptide 5

## Abstract

**Background:**

Without a viable cure, chronic kidney disease is a global health concern. Inflammatory damage in and around the renal tubules dictates disease severity and is contributed to by multiple cell types. Activated in response to danger associated molecular patterns (DAMPs) including ATP, the NOD-like receptor protein-3 (NLRP3) inflammasome is integral to this inflammation. In vivo, we have previously observed that increased expression of Connexin 43 (Cx43) is linked to inflammation in chronic kidney disease (CKD) whilst in vitro studies in human proximal tubule cells highlight a role for aberrant Cx43 hemichannel mediated ATP release in tubule injury. A role for Cx43 hemichannels in priming and activation of the NLRP3 inflammasome in tubule epithelial cells remains to be determined.

**Methods:**

Using the Nephroseq database, analysis of unpublished transcriptomic data, examined gene expression and correlation in human CKD. The unilateral ureteral obstruction (UUO) mouse model was combined with genetic (tubule-specific Cx43 knockout) and specific pharmacological blockade of Cx43 (Peptide5), to explore a role for Cx43-hemichannels in tubule damage. Human primary tubule epithelial cells were used as an in vitro model of CKD.

**Results:**

Increased Cx43 and NLRP3 expression correlates with declining glomerular filtration rate and increased proteinuria in biopsies isolated from patients with CKD. Connexin 43-tubule deletion prior to UUO protected against tubular injury, increased expression of proinflammatory molecules, and significantly reduced NLRP3 expression and downstream signalling mediators. Accompanied by a reduction in F4/80 macrophages and fibroblast specific protein (FSP1^+^) fibroblasts, Cx43 specific hemichannel blocker Peptide5 conferred similar protection in UUO mice. In vitro, Peptide5 determined that increased Cx43-hemichannel activity primes and activates the NLRP3 inflammasome via ATP-P2X7 receptor signalling culminating in increased secretion of chemokines and cytokines, each of which are elevated in individuals with CKD. Inhibition of NLRP3 and caspase 1 similarly decreased markers of tubular injury, whilst preventing the perpetual increase in Cx43-hemichannel activity.

**Conclusion:**

Aberrant Cx43-hemichannel activity in kidney tubule cells contributes to tubule inflammation via activation of the NLRP3 inflammasome and downstream paracrine mediated cell signalling. Use of hemichannel blockers in targeting Cx43-hemichannels is an attractive future therapeutic target to slow or prevent disease progression in CKD.

Video Abstract

**Supplementary Information:**

The online version contains supplementary material available at 10.1186/s12964-023-01245-7.

## Background

Chronic kidney disease (CKD) is caused by the convergence of fundamental mechanisms that underlie age-related tissue dysfunction, including chronic “sterile” NOD-like receptor protein-3 (NLRP3)-induced inflammation [1] and cellular senescence [2]. Activation of the NLRP3 inflammasome and our innate immune response, is a major perpetrator of inflammatory damage in both CKD [3] and cardiovascular disease (CVD) [4], with NLRP3 activation linked to renal injury-induced cardiac dysfunction [5]. Tubulointerstitial fibrosis (TIF) is the key underlying pathology of CKD and develops in response to various morphological and phenotypic changes, including partial epithelial-to-mesenchymal transition (p-EMT), inflammatory cell infiltration, fibroblast activation and extracellular matrix (ECM) remodeling [6]. Culminating in inflammation and fibrosis, these changes sit downstream of cell senescence and its proinflammatory senescence associated secretory phenotype (SASP) [7]. With the tubular epithelium implicated as the primary site of both senescence [8] and inflammation, drugs targeting senescent cells [9] and the NLRP3 inflammasome [10] are receiving increased attention. However, although promising, more information about safety, tolerability and off-target effects of these drugs is required. Consequently, urgent therapeutic approaches are required to alleviate the burden of this debilitating disease.

Studies linking altered connexin (Cx) activity to inflammation, [11] senescence [12] and fibrosis [13] suggest that stabilising hemichannel and/or gap junction mediated intercellular communication (GJIC), may prevent the onset of inflammation across multiple disease states. In the kidney, we previously reported that heterozygous Cx43^+/−^ mice (50% global deletion) exhibit decreased tubular macrophage infiltration, and reduced numbers of active fibroblasts when induced to exhibit interstitial inflammation and fibrosis via unilateral ureteral obstruction (UUO) [14]. Furthermore, loss of Cx43 expression protected against disassembly of the adherens junction, a key trigger in initiation of those events which underlie p-EMT [15]. I*n vitro*, we determined that these effects are mediated by high levels of adenosine tri phosphate (ATP) and downstream purinergic signaling, notably via activation of the P2X7 receptor (P2X7R). Importantly, we determined the source of this ATP to be Cx43 hemichannels, with Cx43 specific hemichannel blocker Peptide 5 protecting against these effects in injured human primary proximal tubule cells [16].

Building on these observations, Xu et al. recently determined that Cx43 knockdown in renal tubules of UUO-mice is paralleled by a reduction in purine metabolites in the urine [17]. Proximal tubules isolated from sham mice had increased intracellular ATP as compared to UUO mice alone, an effect which was dampened when UUO mice were treated with Gap26 (a Cx43 peptide that blocks both gap junction and hemichannel activity). Exogenous application of lipopolysaccharide (LPS) and ATP to bone marrow derived macrophages (BMDMs) stimulated macrophage Gasdermin D cleavage, pyroptotic events triggered in response to canonical (caspase 1) or non-canonical (caspase 4,5) inflammasome activation and corroborated in vivo by observations in which diminished tubule Cx43 expression paralleled a reduction in the number of Gasdermin D F4/80 positive cells [17]. Interestingly, whilst the studies by Xu et al. link altered tubule Cx43 expression to macrophage pyroptosis, observations across several models of eye disease, associate aberrant Cx43 hemichannel activity to NLRP3 inflammasome activation [18, 19]. Consequently, with the incidence of retinopathy and nephropathy inextricably linked [20], here we utilise an in vivo model of advanced interstitial inflammation and fibrosis and determine that genetic deletion of tubule-specific Cx43 (Pax8-rtTA- cre:cx43 flox Cx43^−/−^) or pharmacological inhibition of Cx43-hemichannel activity using the Cx43 specific hemichannel inhibitor Peptide5 [21], preserves tubule structure, reduces interstitial fibrosis and attenuates NLRP3 inflammasome activity and inflammation in the face of UUO. Importantly, we report a novel and direct role for Cx43-hemichannel mediated activity in both priming and P2X7R-mediated activation of caspase 1 mediated interleukin-1β (IL1β) cleavage. Furthermore, we determine that these events perpetuate sterile inflammation through a vicious feed-back loop in which NLRP3 activity exacerbates Cx43-hemichannel mediated real time ATP release. In assessing downstream implications of this dysregulated cell communication, proteome array analysis determined a role for aberrant Cx43-hemichannel mediated ATP the secretion of pro-inflammatory mediators, effects partially blocked by Peptide5 and evidenced in vivo by reduced macrophage infiltration and fibroblast accrual.

In summary, this study reports that aberrant Cx43-hemichannel mediated ATP release primes and activates the NLRP3 inflammasome and that closing Cx43-hemichannels protects against tubular damage induced by this vicious feedforward cycle of events. With Cx-hemichannel inhibitors already in clinical trial for multiple morbidities of chronic inflammation [22], delineating a role for Cx43-hemichannel activity in the pathogenesis of TIF would undoubtedly open future opportunities for clinical intervention in late-stage kidney inflammation.

## Methods

### Materials

Primary human proximal tubule epithelial cells (hPTECs) and human kidney (HK2) proximal tubule cells were purchased from the American Type Culture Collection (ATCC; LGC Standards), and tissue culture supplies were purchased from Invitrogen (Paisley, UK). Immobilon-Fl PVDF membrane was from Millipore (Watford, UK), whilst Odyssey blocking buffer and secondary fluorescent antibodies were purchased from LI-COR (Cambridge, UK). Antibodies for NLRP3, interleukin-18 (IL18), interleukin-6 (IL6), tumor necrosis factor (TNFα), E-cadherin, N-cadherin, collagen I and collagen IV were purchased from ABCAM (Cambridge, UK). Antibodies for caspase 1 were purchased from Cell Signalling Technology (Danvers, MA, USA), whilst antibodies for fibronectin were obtained from SantaCruz (Santa Cruz, CA, USA). Recombinant human transforming growth factor beta 1 (TGF-β1) and non-hydrolysable ATPγS were obtained from Sigma (Poole, UK), as were all other general chemicals. Peptide5 and scrambled Peptide5 were synthesized by SynPeptide Co (Shanghai, China). NLRP3 inhibitor CY-09, caspase 1 inhibitor AC-YVAD-CMK and P2X7R inhibitor A438079 were obtained from Bio-Techne (Abingdon, UK). SYBR Green and RNeasy mini kits were purchased from QIAGEN (Manchester, UK), whilst a high-capacity complementary (c)DNA reverse transcriptase kit was purchased from ThermoFisher (Loughborough, UK). Primers were synthesised by Invitrogen (Loughborough, UK). ATP/Null biosensors were from Sarissa Biomedical Ltd (Coventry, UK) and fluorodishes from World Precision Instruments (Hertfordshire, UK).

### Animal model – UUO/Pax 8 dox

All procedures regarding whole animal studies were in accordance with the ARRIVE (Animal Research: Reporting In Vivo Experiments) guidelines 2.0 and within the European Union Guidelines for the Care and Use of Laboratory Animals and approved by the local ethics committee of INSERM. Animals were housed at a constant temperature with free access to water and food.

Mice were male and of a C57BL/6 background. Cx43^flox/flox^ mice were purchased from “the EMMA -European Mouse Putant Archive- consortium”. The strain of Cx43 tubular-specific deletion was created as previously described [23]. Briefly, Pax8-rtTA-LC1 were interbred with Cx43flox mice (Pax8-rtTA-LC1/Cx43^flox/flox^) and Cx43 deletion in renal tubular cells was induced by administering 0.2 mg/ml doxycycline in drinking water, containing 2.5% sucrose for 4 weeks starting 1 month after birth. Then, 3–4-month-old mice were subjected to unilateral ureteral obstruction (UUO) as already described [14]. To examine the effect of Cx43 deletion alone, sugar was administered to Pax8-rtTA-LC1/Cx43^flox/flox^ transgenic mice, serving as controls for up to 4 months. Finally, inhibition of Cx43 hemichannels was assessed using Peptide5 intraperitoneal (I.P.) injections twice daily for 9 days (9.3 mg/kg, 24 h after UUO). Peptide5 is a Cx43 specific hemichannel blocker which binds to the extracellular loop of Cx43 [21]. All mice were sacrificed at day 10.

### Renal morphology, evaluation of interstitial fibrosis and immunohistochemistry

Kidneys were fixed in formalin solution (4%) and embedded in paraffin as already described [14]. Sections (4 mm thick) were stained with Masson’s trichrome for histological evaluation. Interstitial fibrosis was assessed semi-quantitatively on Sirius Red stained sections. Fibrosis was then quantified using analysis morphometric software (Olympus) that allowed the formation of a binary image, in which the stained area could be automatically calculated as percentage of the image area. Ten fields per section that covered the entire cortex were randomly selected. Tubular dilation was scored as previously reported [14]. Scoring was performed blind on coded slides. Immunostaining for F4/80 (1:200, Serotec), FSP1 (1:200, Abcam) and NLRP3 (1:200, AdipoGen) was performed on paraffin embedded sections as already described [24]. Immunofluorescence for Cx43 (1:200, Sigma-Aldrich) was performed on methanol fixed cryosections as previously reported [14]. Alexa Fluor secondary antibodies (1:500) were used for detection. Sections were counterstained with Evans Blue. Negative controls included omission of first antibodies or preincubation of first antibodies with immunogenic peptides.

### Human samples

Primary hPTECs were maintained in Renal Epithelial Cell Basal Media from ATCC, supplemented with Renal Epithelial Cell Growth Kit components (PCS-400-040) in a humidified atmosphere at 37 °C with 5% CO_2_. Cells were treated with TGF-β1 or ATPγS ± a 30-minute pre-incubation with Cx43 hemichannel blocker Peptide5 (25µM) or P2X7R antagonist A438079 (50µM − 100µM) for 48 h. For real time ATP biosensing studies, human kidney (HK2) cells (passage 18–30) were maintained in DMEM/Hams F12 medium, supplemented with 10% foetal calf serum, glutamine (2mmol/L) and epidermal growth factor (5ng/mL), in a humidified atmosphere at 37 °C with 5% CO_2_. Cells are proximal tubular epithelial cells, immortalized by the transduction of human papilloma virus 16 (HPV-16) E6/E7 genes and are mycoplasma-free. Cells were seeded in low-glucose DMEM/F12 (5mmol/L) for 48 h and serum-starved overnight prior to treatment with TGF-β1 (2–10ng/mL) for 48 h, ± a 30-minute pre-incubation with Peptide 5 (25 μm), NLRP3 inhibitor CY-09 (1–20µM) or caspase 1 inhibitor AC-YVAD-CMK (0.1–10 µg/mL).

### Nephroseq

We used unpublished publicly available datasets from the Nephroseq database (www.nephroseq.org, University of Michigan, Ann Arbor, MI, USA) to further examine gene expression in both healthy human kidneys and in kidneys from people with CKD. Expression data was obtained from the ‘Nakagawa CKD Kidney’ [25] dataset in which a total of 53 renal biopsies from individuals with CKD (48 in discovery cohort and 5 in validation cohort) and 8 controls (5 in discovery cohort and 3 in validation cohort) were analysed on Agilent Whole Human Genome Microarrays (4 × 44k). Gene expression profiles were analysed to identify genes that are differentially expressed between individuals with CKD versus healthy controls. This dataset was previously named Nakagawa CKD [25]. Correlation of expression to proteinuria was performed using data from the Schmid Diabetes TubInt study [26]. Gene expression in 22 human renal biopsies were analysed on an Affymetrix HG-U133A microarray. The samples include tissues from 3 living donors (controls), 4 people with minimal change disease, 4 cadaveric donors and 11 people with diabetic nephropathies. Sample data includes sex, age, weight, glomerular filtration rate (GFR), and diabetes type, among others. This dataset was previously named Schmid Diabetes [26]. Correlation of gene expression to GFR was performed using data from the Ju CKD 2 dataset [27].

### Total RNA extraction and quantitative real-time PCR

#### In vivo work

Total RNA was extracted from the renal cortex using TRIzol reagent (Euromedex), RNA quality was checked by control of optical density (OD) at 260 and 280 nm. cDNA was synthesized from 1 mg RNA using the Fermentas H Minus First-Strand cDNA Synthesis Kit according to the manufacturer’s instructions. Quantitative PCR experiments were performed as previously described [18]. Each sample was run in triplicate, and analysis of relative gene expression was done using the 2 ^−ΔΔCT^ method. Results are expressed in graphs as arbitrary units, which represent the ratio of the target gene to the internal control gene hypoxanthine phosphoribosyl transferase (*HRPT*). Sequences of primers used in our studies are listed in Table 1.

**Table 1:** Mouse primers used

Gene	Forward Primer	Reverse Primer
IL1β	CCACAGACCTTCCAGGAGAATG	GTGCAGTTCAGTGATCGTACAGG
IL6	GCTACCAAACTGGATATAATCAGGA	CCAGGTAGCTATGGTACTCCAGAA
MCP1	GGCTGGAGAGCTACAAGAGG	CTCTTGAGCTTGGTGACAAAAA
NGAL	CCATCTATGAGCTACAAGAGAACAAT	TCTGATCCAGTAGCGACAGC
KIM1	TCAGATTCAAGTCTTCATTTCAGG	CCCCCTTTACTTCCACATAAGAA
Cx43	GTGCCGGCTTCACTTTCA	GGAGTAGGCTTGGACCTTGTC
VCAM	TGGTGAAATGGAATCTGAACC	CCCAGATGGTTTCCTT
HPRT	GGAGCGGTAGCACCTCCT	CTGGTTCATCATCGCTAATCA

### Primary hPTEC RNA

RNA was extracted using an RNeasy mini kit (QIAGEN), reverse transcribed (Invitrogen) and subjected to quantitative real-time PCR (SYBR GreenER, Invitrogen) using a StepOne Plus instrument (Applied Biosystems Inc, Foster City, CA). RNA and cDNA concentrations were measured using a Nanodrop. cDNA expression of candidate genes was obtained by relative comparison to a standard curve of serially diluted cDNA. Primers can be found in Table 2. Dissociation (melt) curve analysis confirmed primer specificity and checked for potential contamination.

**Table 2:** Human primers used

Gene	Forward Primer	Reverse Primer
NRLP3	ggactgaagcacctgttgtgca	tcctgagtctcccaaggcattc
IL1β	ccacagaccttccaggagaatg	gtgcagttcagtgatcgtacagg
IL18	gatagccagcctagaggtatgg	ccttgatgttatcaggaggattca
GAPDH	gtctcctctgacttcaacagcg	accaccctgttgctgtagccaa

### Western blotting

Protein isolation, separation by SDS-gel electrophoresis and transfer onto Immobilon-Fl PVDF membranes was performed as described previously [28]. Membranes were blocked with Odyssey blocking buffer (LI-COR). Specific polyclonal antibodies were used to probe for NLRP3 (1:500), IL18 (1:1000), IL1β (1:500), IL6 (1:1000), TNFα (1:1000), caspase 1 (1:500), fibronectin (1:2000), collagen I (1:1000), collagen IV (1:1000), E-cadherin (1:500), and N-cadherin (1:1000) in human lysate. Bands were visualized using an OdysseyFC and semi-quantified using ImageStudio (v5.2, LI-COR).

### Proteome inflammation array

An inflammation antibody array (RnD systems) assessed TGF-β1 induced regulation of 105 inflammatory markers in primary hPTECs and was performed by following manufacturer’s instructions. Briefly, hPTECs were cultured in low glucose DMEM/F12 (5mM) for 48 h, prior to overnight serum-starvation, then subsequently stimulated for 48 h with TGF-β1 (10ng/ml) ± Peptide5 (25µM). Cell supernatant was collected and incubated overnight with pre-blocked membranes spotted in duplicate with capture antibodies. An 800CW fluorescent streptavidin/biotinylated cocktail mixture was used to visualise expression protein/antibody complexes using an OdysseyFC which was semi-quantified using ImageStudio (v5.2, LI-COR).

### Inflammasome caspase-glo 1 assay

Caspase 1 activity was assessed using the Caspase-Glo-1 Inflammasome Assay (Promega). hPTEC cells were seeded onto a solid, white, clear bottomed 96-well plate. After treatment with appropriate stimuli, the Caspase-Glo 1 reagent was added (1:1), mixed, and left at RT for 1 h. Luminescence was measured using a Hidex Chameleon 4.42 with MikroWin 2000 v4.38 software. For initial optimisation experiments, cells were pre-incubated with the supplied specific caspase inhibitor AC-YVAD-CHO (1µM).

### Real time ATP biosensing

ATP biosensors (Sarissa Biomedical, Coventry UK) were used as described previously [29]. A null biosensor was used to account for non-specific electro-active artefacts. Glycerol was included in all recording solutions at a saturating concentration (2mM). HK2 cells were seeded onto coverslips (10 mm diameter), treated as described above and placed in a chamber containing Ca^2+^-containing BSS perfused at 6ml/min (37 °C). After a 10 min acclimatisation period, ATP and null biosensors were bent and lowered so that the electrode laid parallel and close to the cell monolayer. A calibration peak of known ATP concentration (10µM) was used in each recording to allow for quantification. Recordings were acquired at 4 Hz with a Micro CED (Mark2) interface using Spike (v8.17) software.

### Carboxyfluorescein dye uptake

Primary hPTECs were cultured on fluorodishes (22 mm diameter) in low glucose DMEM/F12 (5mmol/l) for 48 h, followed by overnight serum-starvation and subsequent treatment. For the following steps, a balanced salt solution (BSS, pH 7) was used, comprising of NaCl (137mM), KCl (5.4mM), MgSO_4_ (0.8mM), Na_2_HPO_4_ (0.3mM), KH_2_PO_4_ (0.4mM), NaHCO_3_ (4.2mM), HEPES (10mM) and glucose (5mM). A Ca^2+^-free BSS (zero CaCl_2_ + EGTA (1mM)) plus carboxyfluorescein (200µM) solution was added for 10 min, which was subsequently exchanged with Ca^2+^-containing BSS (1.3mM) plus carboxyfluorescein (200µM) for 10 min, then washed with Ca^2+^-containing BSS (12ml). Images were acquired with a Cool Snap HQ CCD camera (Roper Scientific) and Metamorph software (Universal Imaging Corp., Marlow, Bucks, UK). ImageJ was used to quantify dye uptake by selecting a region of interest (ROI) around each cell (approx. 10–15 cells/dish). Mean pixel intensity was measured, with background fluorescence subtracted [30].

### Statistical analysis

Data are presented as mean ± SEM unless otherwise stated, with further details of statistical analysis provided in relevant figure legends. Analysis was performed using GraphPad Prism v9.4 (GraphPad Software, CA, USA), where statistical significance was calculated using analysis of variance (ANOVA) or T-tests, as appropriate, with Tukey *post-hoc* mean comparisons. Values of *P* < 0.05 were considered significant.

## Results

### Deletion of tubular Cx43 protects against inflammation and interstitial fibrosis following obstructive nephropathy

**Fig. 1:**
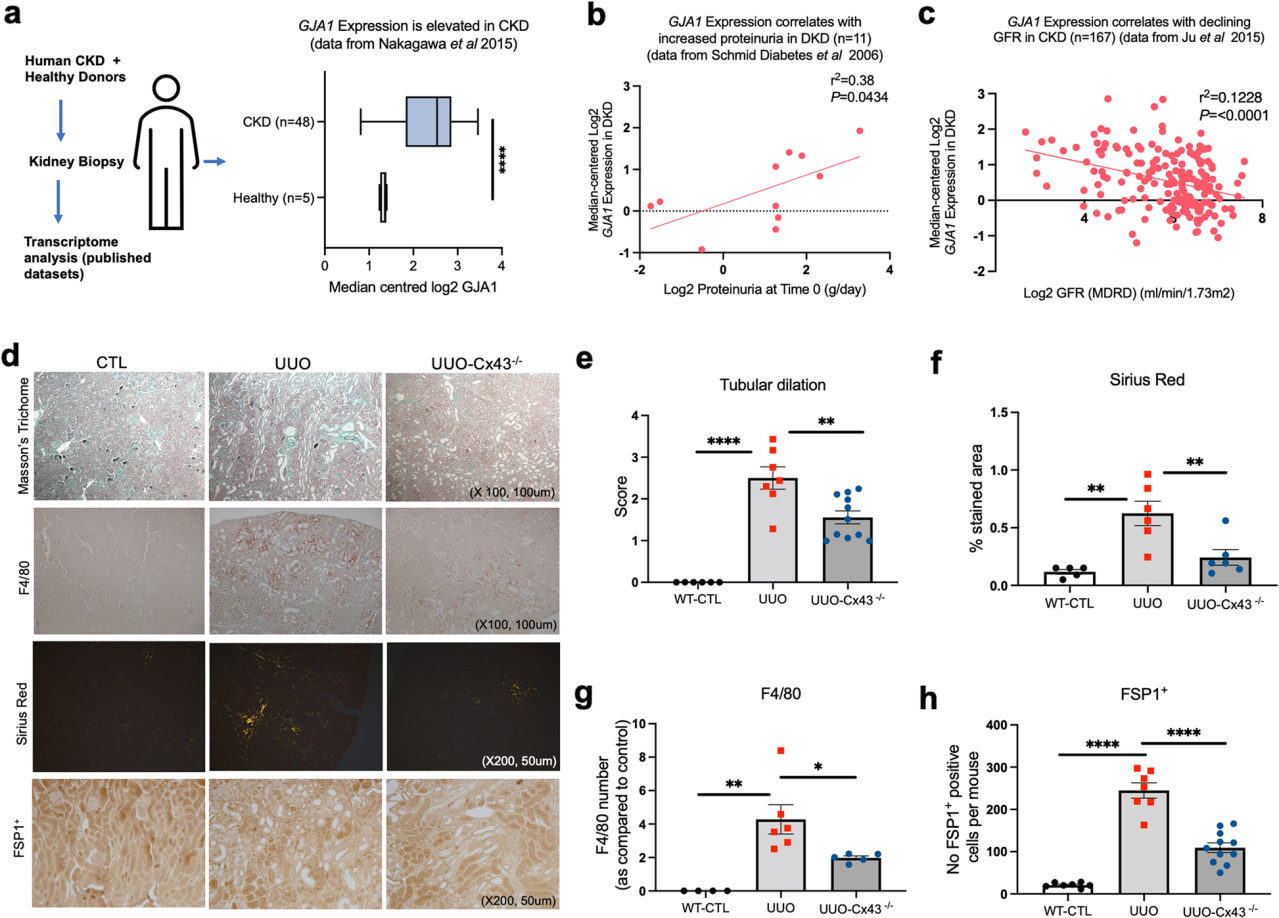
Deletion of Cx43 in the proximal tubules preserves renal structure and protects against inflammation and fibrosis. Datasets published on Nephroseq were examined for Cx43 expression. Analysis of a published transcriptomic dataset shows that *GJA1* expression increases in kidneys from patients with CKD (*n* = 48 with CKD and *n* = 5 normal controls: (**a**) and is positively correlated to proteinuria (**b**) and declining GFR (**c**)^[^. Representative micrographs (**d**) and quantification of Masson’s Trichrome (tubular dilation) (**e**), Sirius Red (interstitial fibrosis) (**f**), F4/80 (macrophage abundance) (**g**) and FSP1^+^ (fibroblast number) (**h**) staining demonstrate that inflammation and fibrosis is significantly ameliorated in UUO-Cx43^−/−^ mice as compared to UUO mice. All groups are *n* = 5–12 unless otherwise specified, with ANOVA and Tukey post-test used for experimental comparisons except for transcriptomic data where an unpaired t-test with Welch’s correction was used. *P < 0.05, **P < 0.01, ***P < 0.001 and ****P < 0.0001

Using human datasets of renal transcriptomic data available on the Nephroseq repository (Fig. 1A-C), [25–27] we assessed the expression of Cx43 in healthy (*n* = 5) and diseased (*n* = 48) kidneys (IgA nephropathy, membranous nephropathy, lupus nephritis, minimal change nephrotic syndrome, membranoproliferative glomerulonephritis, amyloidosis, antineutrophil cytoplasmic antibody-related glomerulonephropathy, diabetic nephropathy and other nephropathies) as encoded by *GJA1* gene expression (Fig. 1a) and correlated this to parameters of renal function (Fig. 1b, c).

*GJA1* expression was increased in diseased kidneys (*n* = 48) versus healthy controls (Fig. 1a; *GJA1*, *P* < 0.0001), where increased *GJA1* expression positively correlates to increased proteinuria (*P <* 0.05) (Fig. 1b) and declining GFR (*P* < 0.0001) (Fig. 1c) in individuals with CKD respectively. With evidence that Cx43 is upregulated in CKD, and that our heterozygous Cx43^+/−^ UUO mouse model exhibits improved renal structure and diminished inflammation [14], we utilised our newly established tubule-directed Pax8-rtTA-cre:cx43 flox Cx43^−/−^ mouse model to determine the effect of Cx43 tubule deletion prior to unilateral ureteral obstruction (UUO). Tubule-directed Cx43^−/−^ deletion was induced by doxycycline prior to UUO (Additional Fig. 1a). Mice were euthanized (*n* = 4–11 per group) and kidneys removed 10 days post UUO prior to preparation for RNA/protein extraction and cryosections using established methodologies (Additional Fig. 1b). qRT-PCR (Additional Fig. 1c) and immunofluorescence (Additional Fig. 1d) determined that Cx43 expression increased in UUO injured kidneys and that tubule expression of Cx43 was ablated in our tubule-directed Cx43^−/−^ UUO compared to wild type (WT) control (*P <* 0.01). Masson’s trichrome (Fig. 1d&e) and Sirius red staining (Fig. 1d&f) demonstrated increased tubular dilation (*P <* 0.0001) (Fig. 1e) and interstitial fibrosis (*P <* 0.01) (Fig. 1f) respectively in UUO mice at 10 days compared to WT control animals, effects significantly reduced in the Cx43^−/−^ model post injury (*P* < 0.01). Moreover, F4/80^+^ macrophage infiltration (*P* < 0.01) (Fig. 1d&g) and increased numbers of FSP1^+^ fibroblasts (*P* < 0.0001) (Fig. 1d&h) were detected in the interstitium 10 days post UUO, whereas deletion of tubule Cx43, reduced macrophage infiltration (*P* < 0.05; Fig. 1g) and fibroblast number (*P* < 0.0001; Fig. 1h) by approximately 60%.

### Inhibition of tubule Cx43 in obstructive nephropathy is paralleled by reduced NLRP3 inflammasome and expression associated markers of tubular injury

**Fig. 2:**
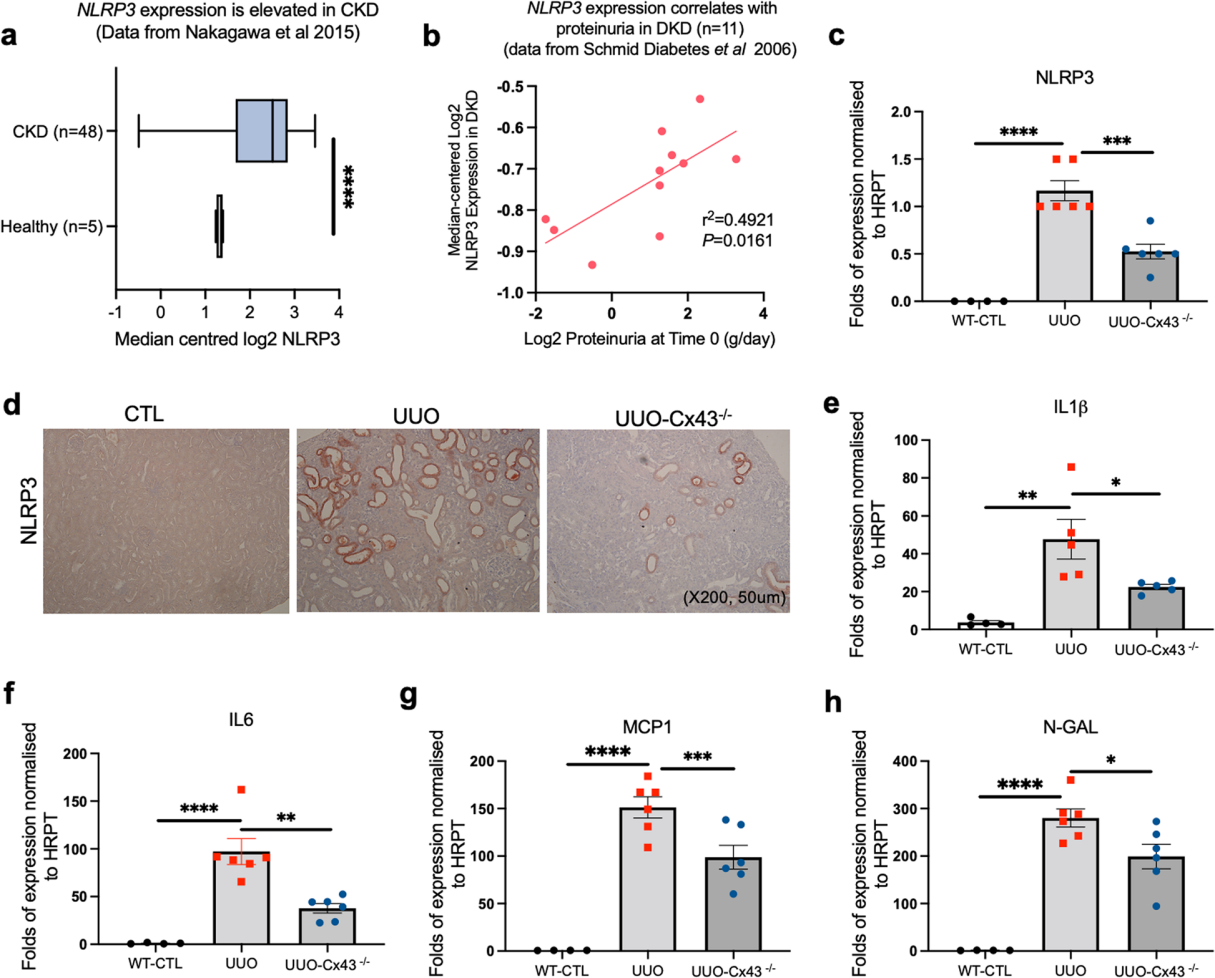
Expression of the NLRP3 inflammasome and markers of tubular injury are reduced in the UUO-Cx43^−/−^mouse. Analysis of a published transcriptomic dataset shows that NLRP3 expression increases in kidneys with CKD (*n* = 48 patients) as compared to healthy controls (*n* = 5) (**a**) and that this positively correlates with proteinuria (**b**) in people with diabetic kidney disease (DKD), the leading cause of end stage renal failure in which tubulointerstitial fibrosis is the key underlying pathology. With evidence that Cx43 and NLRP3 are upregulated in CKD, combined with data that the heterozygous Cx43^+/−^ UUO mouse exhibits improved renal structure and diminished inflammation, we utilised our Pax8-rtTA-cre:cx43 flox Cx43^−/−^ model to determine if Cx43 plays a role in regulating expression of NLRP3 and principal mediators of inflammation. Quantitative PCR (qPCR) analysis and immunostaining of the renal cortex showed a marked downregulation of NLRP3 messenger RNA (mRNA) (**c**) and protein expression (**d**) in UUO-Cx43^−/−^ mice as compared with WT UUO. Similarly, deletion of Cx43 from the proximal tubules prior to induction of UUO protected against an upregulation of IL1β (**e**), IL6 (**f**) and downstream NLRP3 inflammatory mediators, monocyte chemoattractant protein (MCP1) (**g**) and injury marker neutrophil gelatinase-associated lipocalin (N-GAL) (**h**). An ANOVA and Tukey post-test were used for experimental comparisons except transcriptomic data where an unpaired t-test with Welch’s correction &/or a simple linear regression and Pearson’s correlation analysis was used. *P < 0.05, **P < 0.01, ***P < 0.001 and ****P < 0.0001

The NLRP3 inflammasome is a principal mediator of sterile inflammation in the kidney, culminating in caspase 1 mediated cleavage of IL1β and downstream IL6 release. Canonical activation is integral in multiple chronic conditions and targeting its activity is of considerable therapeutic interest. The NLRP3 inflammasome promotes recruitment and activation of resident and infiltrating immune cells [31]. Our tubule-directed Cx43^−/−^ UUO mouse demonstrated reduced macrophage and fibroblast accumulation post UUO, yet a link between tubule Cx43 hemichannel activity and NLRP3 activation remains to be reported. Analysis of renal transcriptomic data from the Nephroseq repository [25–27], determined that NLRP3 (Fig. 2a) and its binding partner apoptosis-associated speck-like protein containing a CARD (ASC) (Additional Fig. 2) were increased in diseased kidneys (*n* = 48; *P <* 0.0001), with NLRP3 expression positively correlated to proteinuria (*P <* 0.05) (Fig. 2b).

Use of qRT-PCR (Fig. 2c) and immunostaining (Fig. 2d) revealed a reduction in NLRP3 expression in our tubule-directed Cx43^−/−^ UUO model (*P <* 0.0001). Similarly, we saw a decrease in IL1β (*P* < 0.05) messenger (m)RNA by approximately 50–60% (Fig. 2e), whilst downstream IL6 (*P <* 0.01) (Fig. 2f), chemokine monocyte chemoattractant protein (MCP1) (*P <* 0.001) (Fig. 2g), and inflammatory marker neutrophil gelatinase-associated lipocalin (NGAL) (*P <* 0.05) (Fig. 2h) also decreased.

In summary, we observed less tubule damage, tubulointerstitial inflammation, and fibrosis in our Pax8-rtTA-cre:cx43 flox Cx43^−/−^ mouse model post UUO, as compared to the Cx43^+/+^ UUO mouse, confirming an essential role for tubular Cx43 expression in renal injury. Moreover, tubule Cx43 expression appears to play a significant role in expression of NLRP3 and downstream mediators IL1β and IL6, with decreased monocyte infiltration, FSP1^+^ cell accrual in the interstitium and reduced expression of MCP1 and NGAL suggesting a clear role for Cx43 expression and function in paracrine mediated signaling.

### Blocking Cx43 hemichannels improves tubular structure, decreases inflammatory cell burden, and reduces interstitial fibrosis

**Fig. 3:**
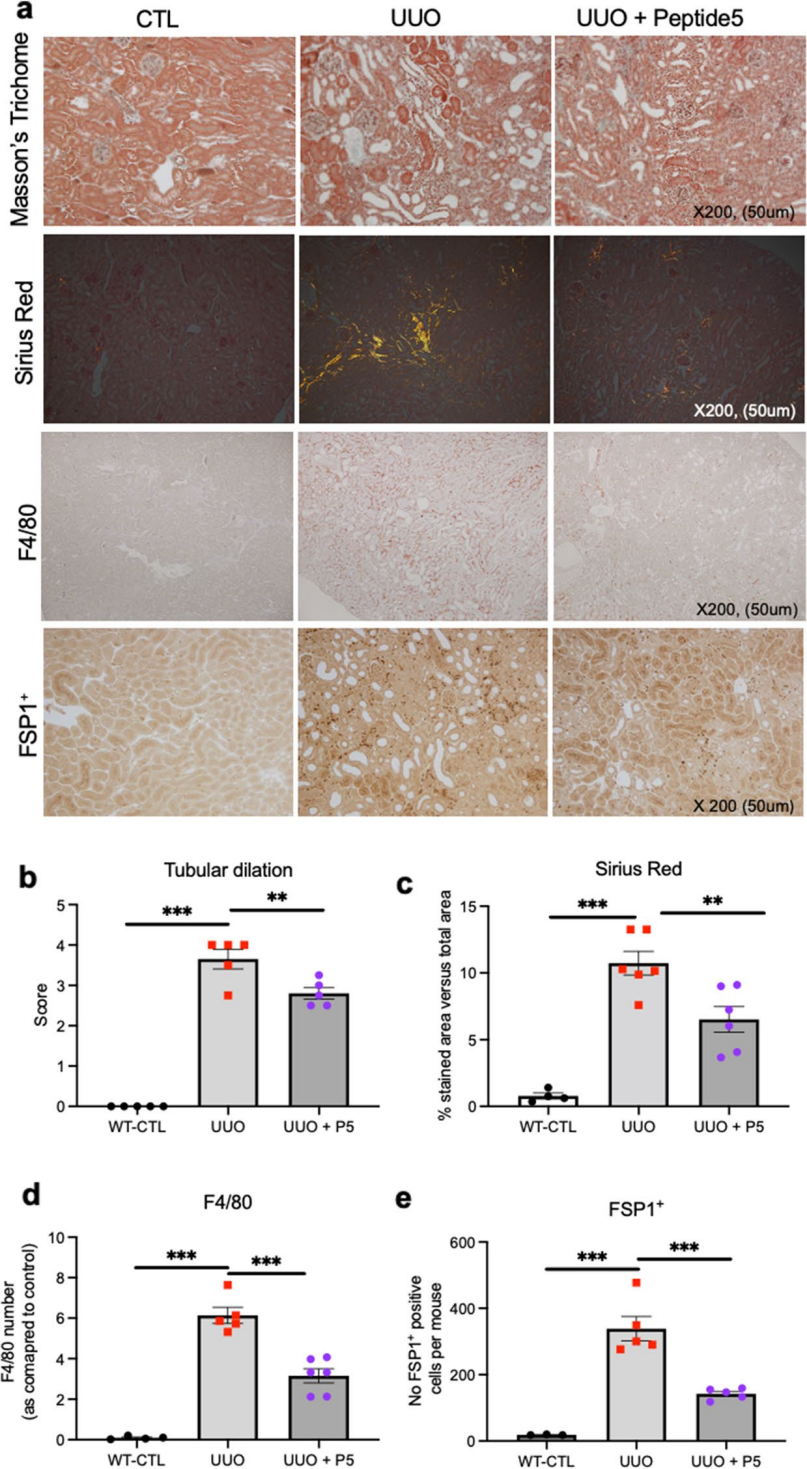
Targeting Cx43 hemichannels protects kidney proximal tubules from interstitial fibrosis, monocyte infiltration and fibroblast activation following obstructive nephropathy. Having ascertained that targeted deletion of Cx43 from the proximal tubules protects against damage from UUO, we determined a role for Cx43 hemichannels in driving tubular damage using the Cx43 hemichannel blocker Peptide5, administered via I.P. injections twice daily for 9 days (9.3 mg/kg, 24 h after UUO). All mice were sacrificed at day 10. Representative micrographs (**a**) and quantification of Masson’s Trichrome (tubular dilation) (**b**), Sirius Red (interstitial fibrosis) (**c**), F4/80 (macrophage infiltration) (**d**) and FSP1^+^ (fibroblast number) (**e**) staining demonstrate that inflammation and fibrosis is significantly ameliorated in the UUO mouse when Peptide5 is administered. All groups are *n* = 4–6 with an ANOVA and Tukey post-test used for experimental comparisons. **P < 0.01 and ***P < 0.001

There are no in vivo studies which have yet employed a Cx43-specific hemichannel blocker to assess a direct role for Cx43 hemichannels in exacerbating the pathology of renal tubulointerstitial inflammation and fibrosis. Peptide5 is a 12 amino acid peptide which targets the 2nd extracellular loop of Cx43 [21] and has been successful in blocking Cx43 hemichannels when delivered topically, intraocularly [32], into cerebrospinal fluid and systemically [21]. Multiple approaches have been used to confirm target applicability and specificity and all have yielded similar and significant benefits across different injury models. Consequently, we used Peptide5 to unravel a novel in vivo role for aberrant Cx43 hemichannel activity in tubular injury. Inhibition of Cx43 hemichannels was achieved using I.P. injections twice daily for 9 days. All mice (*n* = 4–6 per group) were sacrificed at day 10 (Additional Fig. 3a). Mice were euthanized and kidneys were removed 10 days post UUO and prepared for RNA/protein extraction and cryosections using established methodologies (Additional Fig. 3b). Pharmacological intervention of UUO at day 10 with Peptide5 was reno-protective, with tubular dilation (Masson’s Trichome; Fig. 3a&b) reduced from 3.65±0.24 to 2.8±0.14 (*P* < 0.01), whilst interstitial fibrosis (Sirius red; Fig. 3a&c) decreased from 10.73 ±0.89 to 6.18±0.88 (*P* < 0.01). Immunostaining with an F4/80 (Fig. 3a&d) and FSP1^+^ (Fig. 3a&e) antibody demonstrated that inhibition of Cx43-hemichannels precedes a reduction in macrophage infiltration (*P* < 0.001) and fibroblast accumulation (*P* < 0.001).

### Peptide5 blocks UUO-induced increases in NLRP3 and expression of associated mediators of inflammatory damage

The previous results provide compelling evidence that Cx43 hemichannels are integral to renal injury, activity of which is linked to the regulation of inflammatory pathways. As a principal mediator of sterile inflammation, the NLRP3 inflammasome is activated in response to various danger associated molecular patterns (DAMPs) e.g., adenosine triphosphate (ATP), uric acid, and reactive oxygen species (ROS). Using real time ATP biosensing, our previous in vitro studies reported that aberrant Cx43 hemichannel activity is paralleled by increased ATP release in primary tubule cells [15, 33]. Studies in retinal epithelial cells confirm that connexin-mediated hemichannels have an important part to play in NLRP3 inflammasome activation and propagation, with ATP self-inducing its own release and expanding activation of purinergic receptors on infiltrating macrophages [34]. Since mice with the tubule-directed Cx43^−/−^ deletion have decreased expression of NLRP3 and associated downstream signalling mediators (IL1β, IL6, MCP1 and NGAL) we evaluated a role for aberrant Cx43 hemichannel activity as a mechanism for upstream regulation of these proteins.

**Fig. 4:**
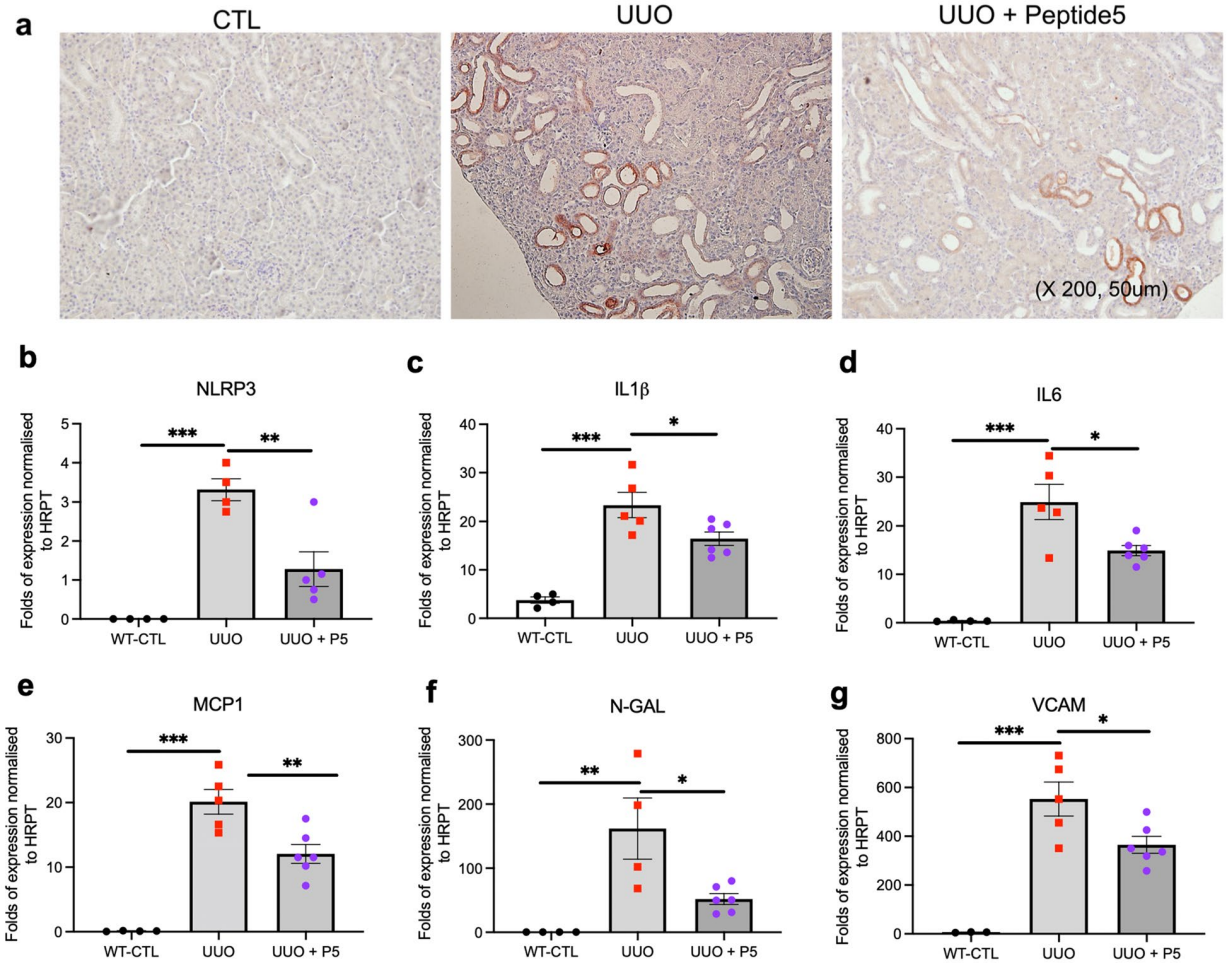
Blocking Cx43-hemichannels in the UUO mouse provides anti-inflammatory protection by reducing NLRP3 inflammasome mediators and downstream drivers of inflammatory burden. Deletion of Cx43 from the proximal tubules protects against the UUO induced upregulation of NLRP3 and associated signalling mediators. To determine if this protection stems from a reduction in Cx43 hemichannel mediated signalling, we administered Peptide 5 via I.P. injections twice daily for 9 days, (9.3 mg/kg, 24 h after UUO) ahead of evaluating the impact that this has on NLRP3 and associated proteins. Immunostaining of the renal cortex and quantitative PCR (qPCR) analysis showed a marked downregulation in NLRP3 protein expression (**a**) and messenger RNA (mRNA) (**b**) in UUO mice treated with Peptide5 as compared with WT UUO. Similarly, blocking Cx43 hemichannels protected against an upregulation of IL1β (**c**), IL6 (**d**) and downstream NLRP3 inflammatory mediators, monocyte chemoattractant protein (MCP1) (**e**) and injury markers neutrophil gelatinase-associated lipocalin (N-GAL) (**f**) and vascular cell adhesion molecule (VCAM) (**g**). All groups are *n* = 4–6 with ANOVA and Tukey post-test used for experimental comparisons. *P < 0.05, **P < 0.01, and ***P < 0.001

Immunostaining of UUO renal cortex demonstrated increased tubular expression of NLRP3 (Fig. 4a), an observation paralleled by elevated NLRP3 (*P <* 0.001), IL1β (*P <* 0.001) and IL6 (*P <* 0.001) mRNA expression (Fig. 4b-d) in lysates of ligated kidneys over a 10-day time course compared to baseline WT control. Expression was reduced when UUO mice were treated with Cx43 hemichannel blocker Peptide5 (NLRP3 (*P <* 0.01), IL1β (*P <* 0.05) and IL6 (*P <* 0.05)). Consistent with the reduction in inflammatory infiltrates observed in our tubule-directed Cx43^−/−^ UUO mice (Fig. 3e), expression of MCP1 (Fig. 4e), NGAL (Fig. 4f) and vascular cell adhesion molecule (VCAM) (Fig. 4g) were upregulated in UUO but decreased by approximately 40% (*P <* 0.01), 70% (*P <* 0.05) and 35% (*P <* 0.05) respectively when Cx43-hemichannel activity was blocked.

### Aberrant Cx43-hemichannel activity primes and activates the NLRP3 inflammasome in hPTECs

Assembly and activation of the NLRP3 inflammasome is a two-step process. Priming (step 1) is initiated in response to NFkB mediated transcriptional control of NLRP3, the apoptosis-associated speck-like protein containing a CARD (ASC), IL1β (rate-limiting step) and IL-18. Activation (step 2) is mediated by ATP binding to the P2X7 receptor. These events culminate in NLRP3 complex assembly, activation of caspase 1 and caspase 1 mediated cleavage of pro-IL1β and pro-IL18 into their mature forms and trigger increased secretion of TNFα and IL6.

**Fig. 5:**
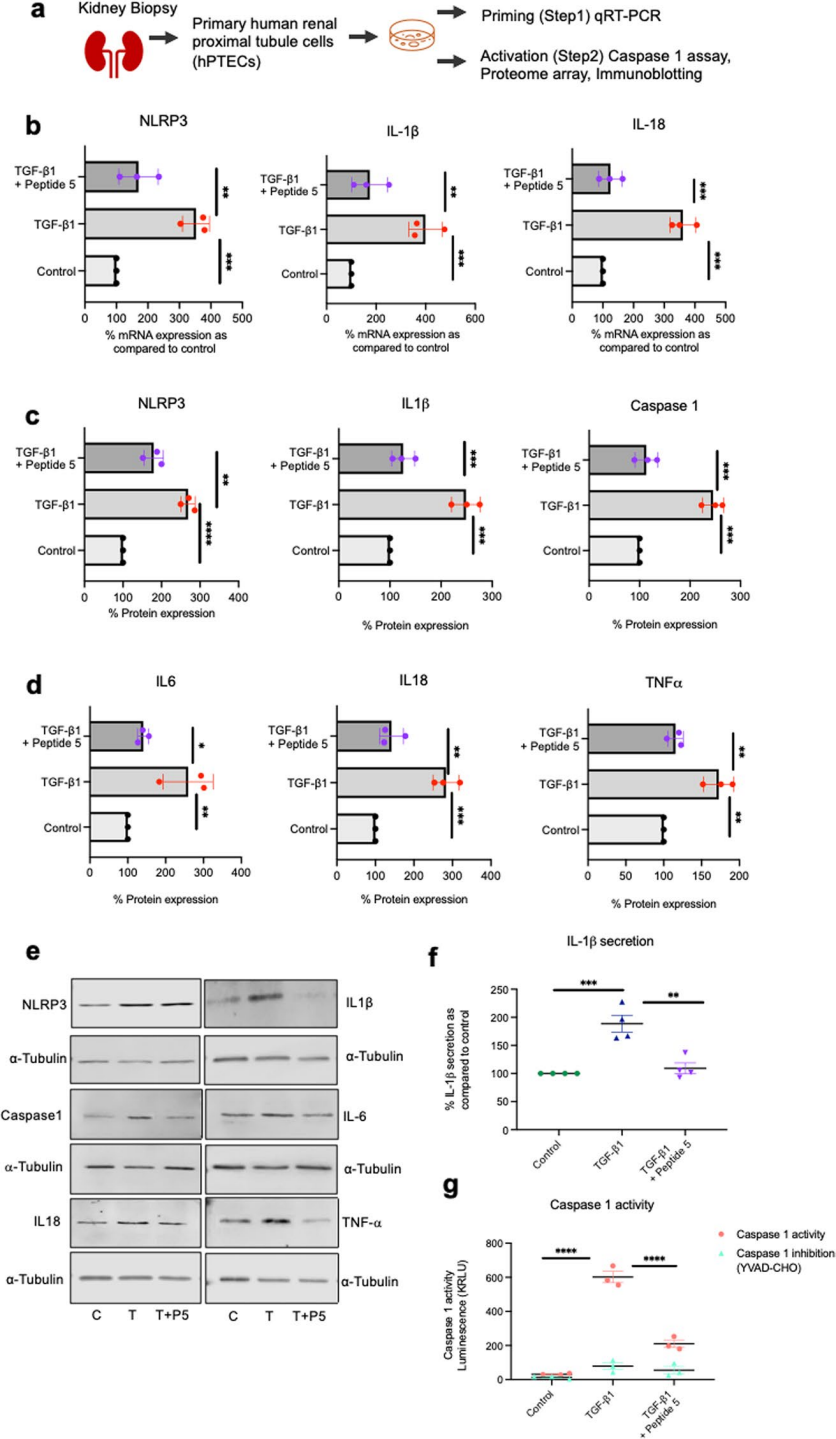
Peptide5 blocks Cx43-hemichannels to inhibit priming and activation of the NLRP3 inflammasome in primary tubule epithelial cells. Primary hPTECs were treated with pro-fibrotic cytokine TGF-β1 (10 ng/ml) for 48 h in the presence/absence of Peptide5 (25 µM) (**a**). qRT-PCR analysis of IL-1β, NLRP3 and IL18 mRNA (**b**) determined a role for Cx43 hemichannel activity in regulation of priming (step 1). Western blot analysis (**c**-**e**), IL1β secretion (**f**) and caspase 1 activity (**g**) determined an upstream role for Cx43-hemichannel activity in NLRP3 inflammasome activation. These data corroborate our in vivo observations and further suggest that aberrant Cx43 hemichannel activity sits upstream of NRLP3 inflammasome activation. All groups are *n* = 3–4. An ANOVA and Tukeys post-test was used for all analysis. *P < 0.05, **P < 0.01, ***P < 0.001 and ****P < 0.0001

Connexin hemichannels open in response to injury and release DAMPs into the intercellular space. Linked to inflammation and fibrosis, ATP is an established DAMP that triggers activation of the P2X7R [35]. Having observed in vivo that Peptide5 blocks Cx43-hemichannels to prevent UUO induced changes in interstitial inflammation and fibrosis, we used hPTECs to evaluate a specific role for tubule epithelial Cx43-hemichannel mediated activity in NLRP3 priming and activation (Fig. 5a). Consistent with the inflammatory response observed after UUO, qRT-PCR (Fig. 5b) determined that a TGF-β1 induced increase in NLRP3, IL1β, and IL18 mRNA was significantly decreased when cells were pre-incubated with Peptide5 (IL1β (*P <* 0.01), NLRP3 (*P <* 0.01) and IL18 (*P <* 0.001)). Immunoblotting of primary tubule lysates determined that Peptide5 decreased the TGF-β1 induced increase in protein expression of NLRP3, IL1β, caspase 1 (active p20 subunit) (Fig. 5c&e), IL6, IL18, and TNFα (Fig. 5d&e). Importantly, Peptide5 also reduced TGF-β1 induced increases in IL-1β secretion (*P <* 0.01; Fig. 5f) and caspase 1 activity (*P <* 0.0001; Fig. 5g). These data confirm for the first time a specific role for Cx43 hemichannel mediated activity in caspase 1 mediated cleavage of IL-1β.

### Connexin 43-hemichannels perpetuate inflammation by an ATP-P2X7R autocrine feedback loop in the inflammasome/inflammation cycle

**Fig. 6:**
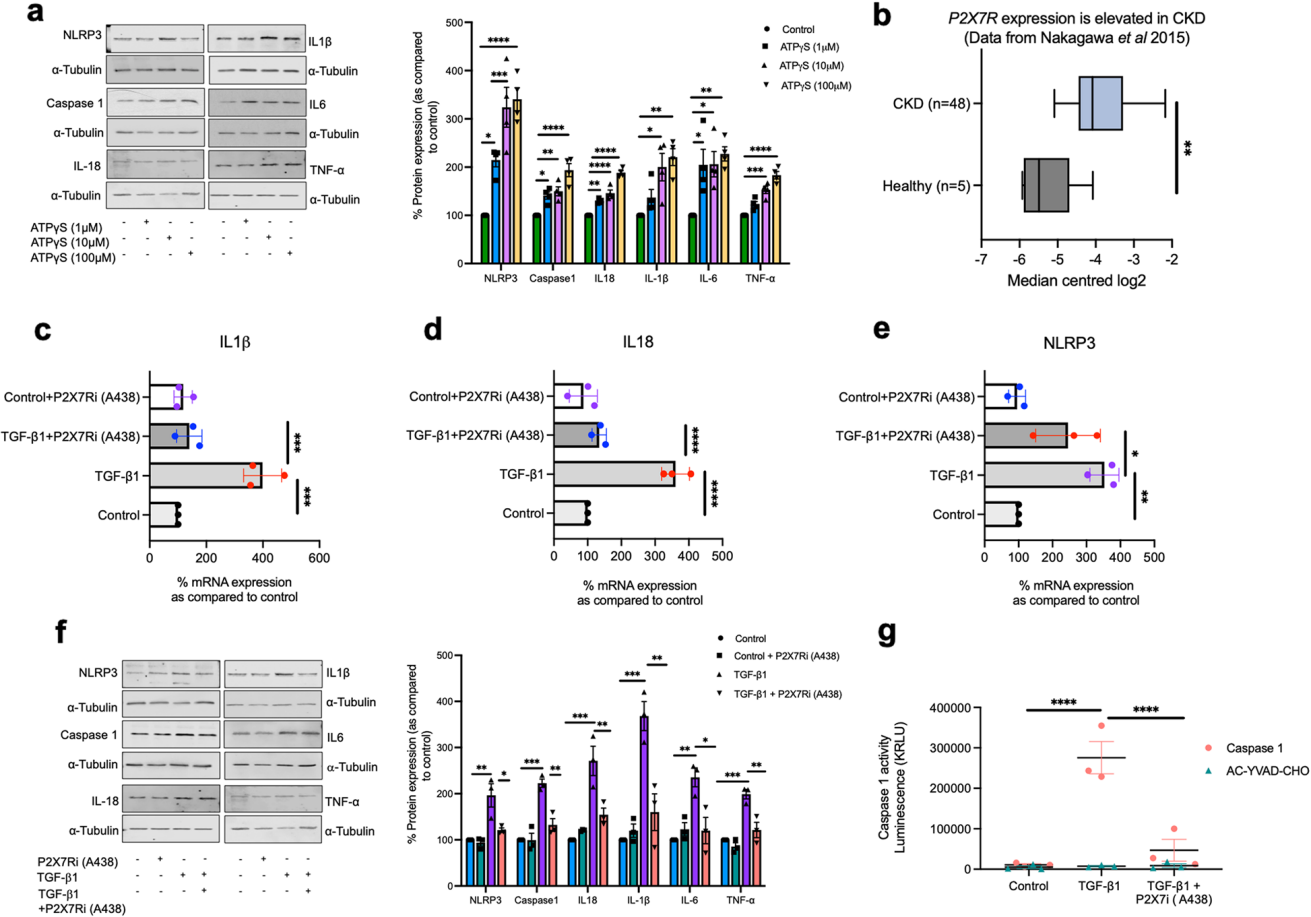
Aberrant Cx43 hemichannel activity regulates NLRP3 priming and activation via P2X7R in hPTECs. To investigate if NLRP3 priming/activation is driven by a Cx43 mediated ATP-P2X7R signalling axis, we established a role for non-hydrolysable ATPγS in increasing expression of the NLRP3 inflammasome and associated downstream mediators, e.g., IL1β, IL6, caspase 1 and caspase 1 (**a**). Analysis of published transcriptome datasets determined that P2X7R is upregulated in patients with CKD (**b**) (*n* = 48 patients). Evidenced by increased IL1β (**c**), IL18 (**d**), and NLRP3 transcription (**e**), qRT-PCR analysis determined that treatment of hPTECS with TGF-β1 induced NLRP3 priming. Inflammasome activation was blunted when TGF-β1 treated hPTECS were pre-incubated with a P2X7R antagonist (A438079), as evidenced by a reduction in protein expression of NLRP3 signalling mediators (**f**) and caspase 1 activity (**g**). All groups are *n* = 3–4. ANOVA and a Tukey post-test was used for experimental comparisons except for transcriptomic data where Welch’s correction was used. *P < 0.05, **P < 0.01, ***P < 0.001 and ****P < 0.0001

We previously determined that hPTECs release ATP, events blocked in the presence of Cx43 hemichannel blocker Peptide5 and P2X7R antagonist A438079 [15]. In Fig. 5 we linked aberrant Cx43-hemichannel activity to both priming and activation of the NLRP3 inflammasome. To determine if these events are likely to be driven by Cx43 hemichannel mediated ATP release, we used non hydrolysable ATPγS (1-100 µM) to determine if ATP could increase expression of key NLRP3 inflammasome mediators IL-1β, Caspase 1, IL-6, IL-18 and TNF-α in primary proximal tubules cells (Fig. 6a). These experiments were preformed ahead of evaluating if P2X7R mediated purinergic signaling lies downstream of these effects.

The P2X7R has a low affinity for ATP and is upregulated (*P* < 0.01) in diseased kidneys of people with CKD (*n* = 48) as compared to healthy control kidneys (*n* = 5) (Fig. 6b). Interestingly, co-incubation of TGF-β1 treated cells with a P2X7R  antagonist, reduced IL1β (*P* < 0.001; Fig. 6C), IL18 (*P* < 0.0001; Fig. 6D) and NLRP3 mRNA expression (*P* < 0.05; Fig. 6e) whilst decreasing NLRP3, caspase 1 (p20 subunit), IL18, IL1β, IL6 and TNFα protein expression (Fig. 6f). Furthermore, P2X7R inhibition significantly reduced the TGF-β1 induced Cx43-hemichannel mediated increase in caspase 1 activity (Fig. 6g).

Previous studies identified that Cx43 gene transcription is regulated by nuclear factor kappa B (NFκB) binding to the Cx43 promoter [36], with Cx43 hemichannel opening regulated in response to a variety of pathophysiological stimuli, including increased intracellular calcium ([Ca^2+^]_i_) and high levels of inflammatory cytokines [37, 38]. Consequently, having established a role for Cx43 hemichannel activity in priming and activation of NLRP3, we hypothesized that a feedback loop exists in which ATP released from Cx43 hemichannels, perpetuates inflammation through its ability to indirectly increase both Cx43 expression and Cx43 hemichannel activity as part of NLRP3 priming and activation.

**Fig. 7:**
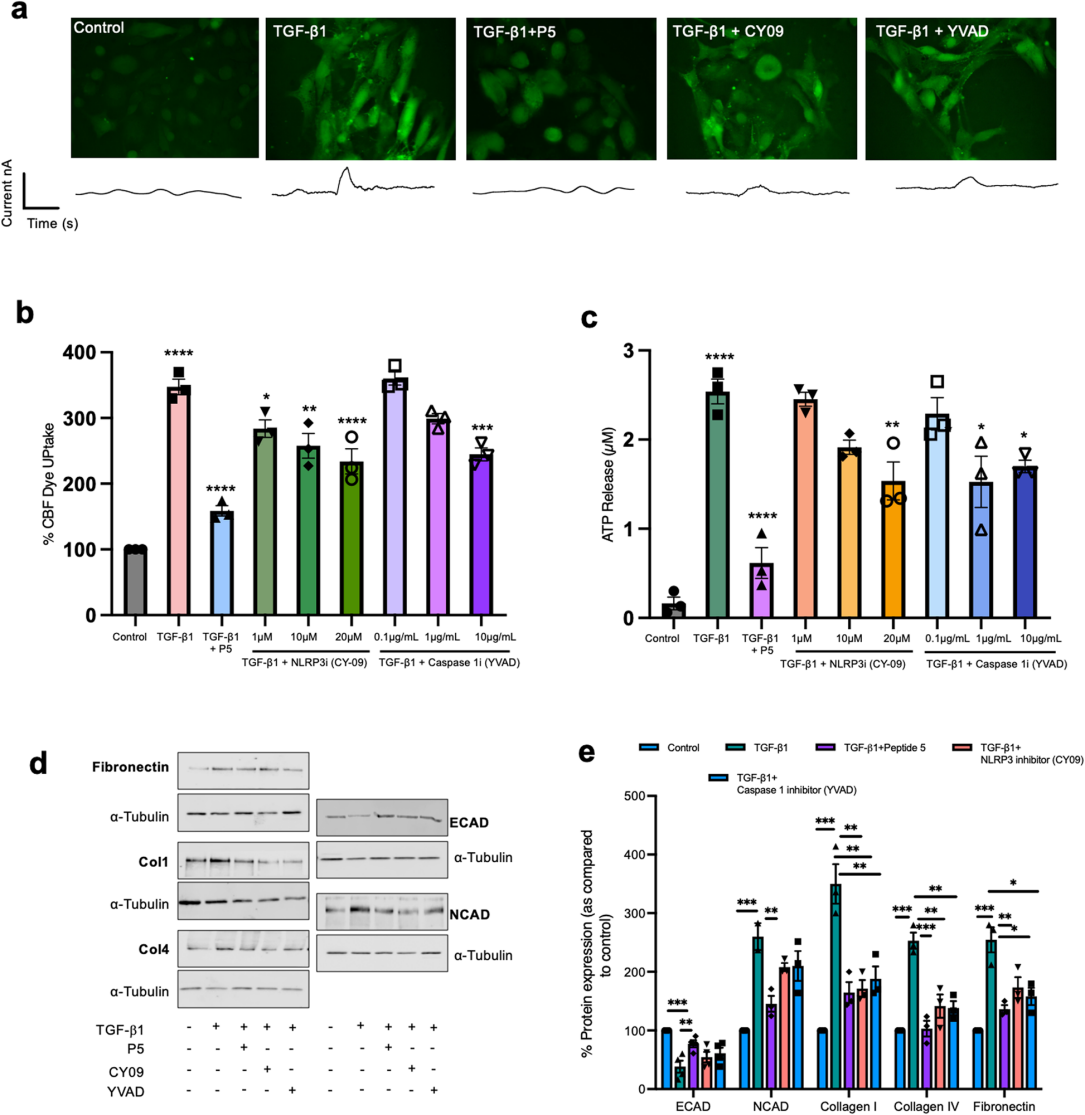
Connexin 43-hemichannels amplify and perpetuate inflammation by an ATP-P2 × 7R autocrine feedback loop. With priming of the NLRP3 inflammasome linked to increased Cx43 expression and inflammation a known gating stimulus of connexin hemichannels, we determined the presence of a feedback loop in which Cx43-NLRP3 activation perpetuates inflammation by an ATP-P2 × 7R autocrine feedback loop. We assessed Cx43 hemichannel mediated carboxyfluorescein dye uptake (**a**&**b**) and real time ATP release (**a**&**c**) in TGF-β1 treated cells which had been pre-incubated with inhibitors to either NLRP3 (CY-09) or caspase 1 (AC-YVAD-CMK). Inhibition of the NLRP3 inflammasome decreased hemichannel mediated dye uptake (**a**&**b**) and ATP release (**c**), suggesting that targeting Cx43-hemichannels offers a potential therapeutic strategy to break the cycle of inflammatory events in tubules of the diseased kidney. The implications of blocking these pathways was further assessed by evaluating expression of extracellular matrix proteins and markers of injury in TGF-β1 treated primary tubule cells pre-incubated with either a Cx43 hemichannel (Peptide5), NLRP3 (CY-09) or caspase 1 (AC-YVAD-CMK) inhibitor (**d**&**e**). An ANOVA and Tukeys post-test was used for all analysis, excluding analysis of transcriptomic data where an unpaired t-test with Welch’s correction was used *P < 0.05, **P < 0.01, ***P < 0.001 and ****P < 0.0001

We assessed the impact of blocking NLRP3 inflammasome activation on Cx43 hemichannel activity using carboxyfluorescein dye uptake and real time ATP biosensing (Fig. 7a-c). Cells were stimulated with TGF-β1± pre-incubation with either an NLRP3 (CY-09) or caspase 1 (AC-YVAD-CMK) inhibitor. Peptide5 reduced dye uptake from 374.4±11.86% to 158.6±–7.9%, whilst inhibition of either NLRP3 or caspase 1 decreased uptake (Fig. 7b) to 234±19.2% (*P <* 0.0001; CY-09 (20µM)) and 244±9.7% (*P <* 0.001; AC-YVAD-CMK (10 µg/mL)) respectively.

TGF-β1 increased real time ATP release from 0.16±0.07µM to 2.54±0.13µM (*P* < 0.0001; Fig. 7c), an effect dramatically reduced to 0.62±0.17µM when cells were pre-incubated with Peptide5 (*P* < 0.0001). Whilst this effect was not fully recapitulated in response to pre-incubation with CY-09 or AC-YVAD-CMK, inhibition of NLRP3 and caspase 1 did limit TGF-β1 evoked ATP release to 1.537±0.21µM (*P* < 0.05), and 1.699±0.07µM (*P* < 0.05) respectively. In assessing the implications of blocking Cx43-hemichannel mediated NLRP3 activation, we treated primary hPTECs with TGF-β1 for 48 h and pre-incubated with either Peptide5 (Cx43-hemichannels), CY-09 (NLRP3) or AC-YVAD-CMK (caspase 1) ahead of evaluating expression of ECM proteins (collagen I, collagen IV, and fibronectin) and downstream markers of NLRP3 induced tubular injury (Fig. 7.d&e). Immunoblotting determined that TGF-β1 downregulated expression of the adherens junction protein E-cadherin (*P <* 0.001), whilst increasing collagen I, collagen IV, fibronectin, and N-cadherin (*P <* 0.001). Whilst increased expression of ECM proteins appeared sensitive to both Cx43-hemichannel, NLRP3 and caspase 1 inhibition, E-cadherin and N-cadherin expression was not restored in the face of decreased NLRP3 activity.

### Peptide5 blocks Cx43 hemichannel activity to reduce the secretion of inflammatory mediators from hPTECs

**Fig. 8:**
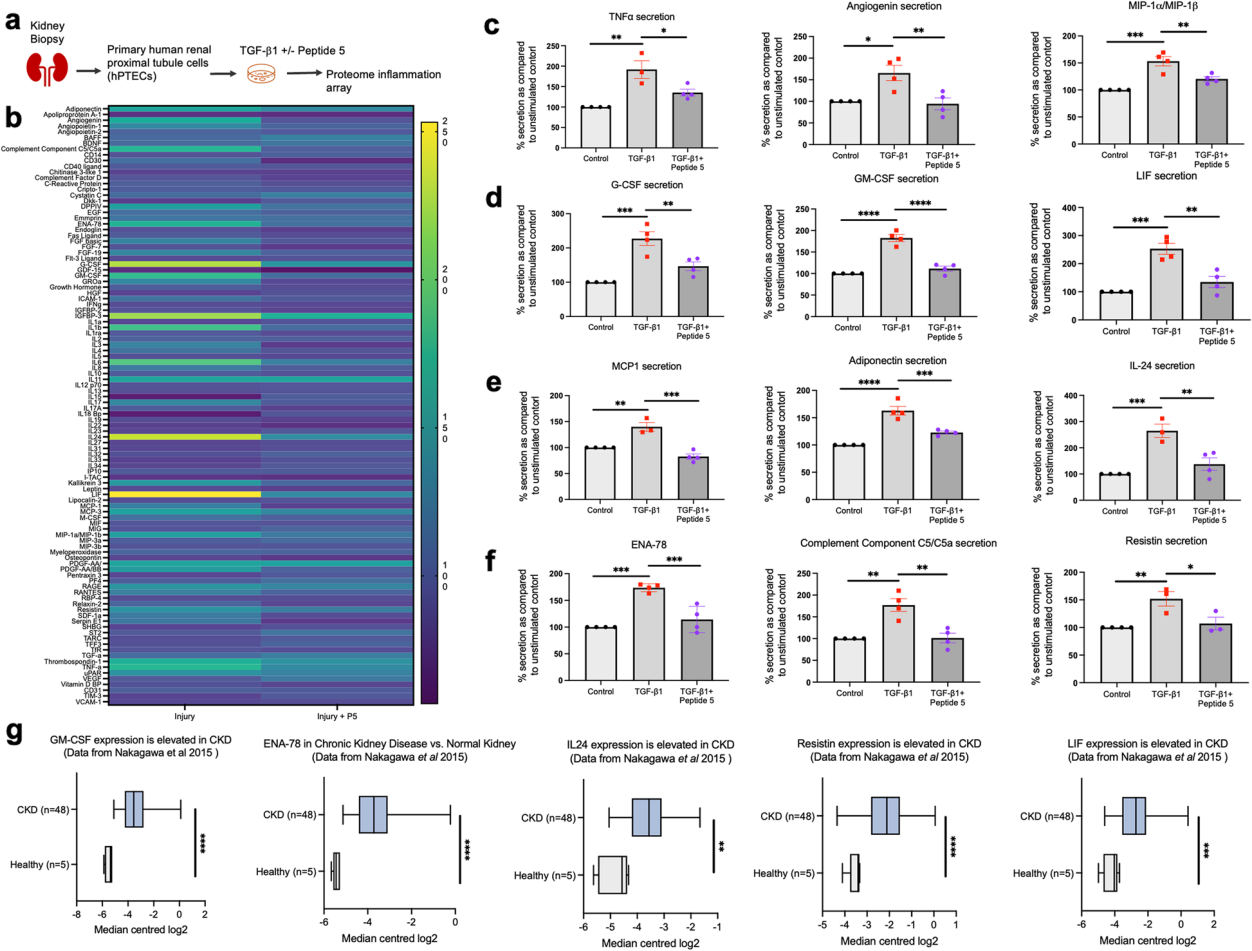
Peptide5 blocks Cx43-hemichannels in primary hPTECs to dampen secretion of the pro-inflammatory secretome. Primary hPTECs were treated with pro-fibrotic cytokine TGF-β1 (10 ng/ml) for 48 h in the presence/absence of Peptide5 (25µM) (**a**) and a proteome profiler inflammation array was used to assess secretion of 105 inflammatory mediators (**b**). Heat map analysis identified multiple changes in protein expression in cells pre-incubated with hemichannel blocker Peptide5 (**b**), with statistical analysis on 12 selected proteins (**c**-**f**) performed. *n* = 4 per group. Extrapolation of this array data using a published transcriptomic dataset on Nephroseq (**g**) shows that granulocyte-macrophage colony-stimulating factor (GM-CSF), epithelial cell-derived neutrophil-activating peptide (ENA-78), interleukin-24 (IL24), resistin and leukemia inhibitory factor (LIF) expression are increased in kidneys of patients with CKD (*n* = 48 in patients with CKD and *n* = 5 normal controls). All groups are *n* = 3–4 unless otherwise specified. ANOVA and Tukeys post-test was used for all proteome array analysis (**c**-**f**), whilst an unpaired t-test with Welch’s correction was used for analysis of transcriptomic data (**g**). *P < 0.05, **P < 0.01, ***P < 0.005, and ****P < 0.001

The NLRP3 inflammasome is linked to immune cell recruitment and fibroblast activation. Activation of the NLRP3 complex is contributed to by Cx43 hemichannel mediated ATP release (Fig. 6). With our in vivo data suggesting that aberrant Cx43 hemichannel activity plays a significant role in paracrine mediated activation and recruitment of F4/80 macrophages and FSP1 positive fibroblasts, proteome profiler arrays were used to establish if Cx43 hemichannel activity triggers the downstream release of soluble paracrine signaling molecules from primary proximal tubule epithelial cells. Cells were treated with profibrotic cytokine TGF-β1 (10ng/mL) ± pre-incubation with Peptide5 (25µM) for 48 h (Fig. 8a). Of 105 cytokines measured (Fig. 8b), we selected representative mediators of CKD-induced inflammation, including MCP1 [39], granulocyte macrophage colony-stimulating factor (GM-CSF), [40] adiponectin [41], TNFα [42], interleukin-24 (IL24) [43], leukaemia inhibitory factor (LIF) [44], and resistin [45] (Fig. 8c-f). Upregulated by TGF-β1, these inflammatory proteins were significantly downregulated when cells were pre-incubated with Peptide5 and Cx43 hemichannel activity blocked. The translational implications of these observations were corroborated using human datasets of renal transcriptomic data (Fig. 8g), where an increase in expression of GM-CSF (*P <* 0.0001), epithelial neutrophil-activating peptide 78 [46] (ENA-78) (*P <* 0.0001), IL-24 (*P <* 0.01), resistin (*P* < 0.0001) and LIF (*P* < 0.001) was observed in diseased kidneys (*n* = 48) versus healthy controls (*n* = 5) based on median centred log2 expression.

## Discussion

Previous studies have identified a link between Cx43 expression and sterile inflammation in multiple age-associated morbidities, including osteoarthritis [47], diabetic retinopathy [18], and age-related macular degeneration [19]. Although Cx43 expression is increased in renal biopsy material from individuals with diabetic nephropathy [15], nephroangiosclerosis [24] and obstructive nephritis [14], here we provide novel evidence that *GJA1* is increased in kidneys from individuals with an array of different types of CKD, and that this increased expression positively correlates with a decline in kidney function, as evidenced by increasing proteinuria and declining GFR. Despite evidencing a connection between Cx43 and functional parameters associated with poor patient outcomes, understanding how to target this damage has been hindered by a paucity of knowledge for how Cx43 mediates its effects.

In the current study, we combined the UUO mouse model with tubule-directed Cx43 knockout (Pax8-rtTA-cre:cx43 flox Cx43^−/−^) and Cx43 blockade (Peptide5) to determine if and how Cx43-hemichannels of the proximal tubule mediate inflammation within the injured tubulointerstitium. The UUO model recapitulates histological and structural changes of advanced CKD and is widely used for studies of senescence and tubular inflammation in models of injury e.g., ageing, CKD, and DN, irrespective of the initiating cause [48]. Ten days post-surgery, we observed increased Cx43 expression, tubular injury, inflammation, and myofibroblast activation with interstitial collagen deposition. Damage was reduced in our tubule-directed Cx43^−/−^ UUO model, as evidenced by diminished tubular injury, decreased macrophage (F4/80) staining, reduced accumulation of FSP1^+^ fibroblasts and interstitial fibrosis. Previous studies report that Peptide5 blocks specifically Cx43-hemichannel mediated ATP release and inhibits activation of purinergic receptors on infiltrating macrophages in a model of retinopathy [49]. In mice treated with Peptide5 24 h prior to UUO, we observed reduced macrophage infiltration, and expression of kidney injury markers e.g., VCAM, MCP1 and NGAL as compared to WT control. Recent studies link increased numbers of pyroptotic macrophages in the UUO mouse interstitium to increased activation of myofibroblasts, events suggested to be driven by the chemokine CXCL10 [17]. Here, we observe that UUO mice with a tubule deletion of Cx43, or treated with Peptide5, exhibit decreased interstitial fibrosis and fibroblast accumulation. In support of these observations, our previous studies highlight a role for tubular Cx43 hemichannels as orchestrators of this response, a statement supported by in vitro studies in which conditioned media transfer (CMT) from kidney tubule cells triggers activation of renal medullary fibroblasts, a response significantly reduced in the presence of Peptide5 [50]. Interestingly, CMT from high glucose/TGF-β1 activated fibroblasts, failed to evoke significant changes in tubule cell phenotype. Whilst our in vivo evidence strongly suggests a role for tubule Cx43 hemichannels in cell-to-cell cross talk, how this aberrant activity links to these downstream deleterious effects remains to be determined.

Soluble chemokines, adhesion molecules and growth factors both recruit and activate infiltrating immune cells [51–53] via paracrine mediated cell communication, inducing multiple pathophysiological pathways e.g., EMT [54] and macrophage-to-myofibroblast differentiation [55]. Consequently, we used proteome array analysis to assess the secretion of 105 proinflammatory mediators from TGF-β1 treated primary proximal tubule cells in the absence and presence of Cx43 hemichannel blocker Peptide5. Co-incubation of TGFβ1 treated hPTECs with Peptide5 reduced the secretion of pro-inflammatory cytokines, with transcriptomic analysis of Nephroseq datasets highlighting their translational implications. Of the many Peptide5-sensitive changes observed, we identified several inflammatory proteins seen in CKD, the action of which are integral to the onset and progression of disease, e.g., granulocyte macrophage colony-stimulating factor (GM-CSF), epithelial neutrophil-activating peptide 78 (ENA-78), interleukin 24 (IL-24), resistin and leukemia inhibitory factor (LIF). In support of our in vivo data, we noted a significant reduction in downstream NLRP3 inflammasome mediators IL1β, TNF-α and IL6. A major perpetrator of inflammatory damage in both acute myocardial infarction and CKD, NLRP3 activation is linked to renal injury-induced cardiac dysfunction [5], and induction of a pro-inflammatory phenotype. Activated in response to various endogenous DAMPs including ATP, increased Cx-hemichannel activity has been linked to NLRP3-mediated inflammation and activation of multiple cell types in various pathologies, including macular degeneration [56] retinopathy [57] and Alzheimer’s [58]. Moreover, elevated plasma NLRP3 and SASP-related inflammatory mediators are associated with an increased risk of cardiovascular events, cardiovascular mortality, and all-cause mortality in patients with CKD, observations supported by the CANTOS (interleukin-1ß) [59] and RESCUE (interleukin-6) [60] trials. As a key mediator of our innate immune response and activated by both DAMPs and pathogen-associated molecular patterns (PAMPs), targeting upstream of sterile DAMP induced inflammation i.e., Cx-hemichannel activity, would leave NLRP3 freely accessible for stimulation by microbial pathogens (PAMPs) and circumvent potential adverse side effects associated with increased risk of infection.

A recent study utilising a UUO mouse model where Cx43 was similarly selectively deleted from the tubules eloquently determined a reduction in protein levels of NLRP3 and caspase 1, supporting our observations linking Cx43 expression with that of NLRP3. Furthermore, the authors associated an increase in macrophage infiltration with high levels of macrophage pyroptosis, an inflammatory form of cell death which can be driven by canonical or non-canonical inflammasome activation. In the absence of confirming caspase 1 activity and caspase 1 mediated cleavage of pro-lL1β, it would be interesting to observe if the pyroptosis was caspase 1 or caspase 4/5/11 dependent, particularly given that the in vitro stimulus was lipopolysaccharide [17]. This earlier study did not explore NLRP3 activation, i.e., caspase 1 activity and IL1β secretion in tubule cells, neither did it link these events to Cx43 hemichannel activity and associated downstream signalling. With the current study specifically focussed on understanding a link between Cx43 hemichannel activity and NLRP3 priming and caspase 1 mediated canonical activation in the kidney tubules, we demonstrate that NLRP3 expression and downstream inflammatory mediators are reduced in tubule cells when Cx43-hemichannel activity is blocked by Peptide5 or tubule specific deletion of Cx43 expression (Pax8-rtTA-cre:cx43 flox Cx43^−/−^). Assembly and canonical activation of the NLRP3 inflammasome is a two-step process, involving initial NFκB mediated transcriptional priming of NLRP3, IL1β (rate-limiting step) and IL18 (Step 1) accompanied by caspase 1 mediated cleavage of pro-IL1β and pro-IL18 into their mature forms (Step 2). We identified that NLRP3 inflammasome priming (mRNA) and P2X7R mediated activation (caspase 1 and IL1β activity) are blunted in TGF-β1 treated hPTECs, when co-incubated with Peptide5. Non-hydrolysable ATPγS was used to assess the efficacy of purinergic stimulation in mediating changes in expression of NLRP3 and its mediators, ahead of evaluating a role for Cx43 hemichannel mediated ATP-P2X7R activation in driving this downstream response. Exhibiting low affinity for ATP, expression of the P2X7R is upregulated in CKD [15] with activation linked to increased NLRP3 activity [61], increased senescence [62, 63] and EMT [64]. Inhibition of P2X7R decreases renal inflammation and improves functional parameters in the Streptozotocin mouse model of type 1 diabetes [65], whilst here, co-incubation of TGFβ1-treated primary hPTECs with a P2X7R antagonist (A438079) prevented NLRP3 activation. Importantly, P2X7R inhibition induced a notable reduction in NLRP3 priming (Step 1), as evidenced by decreased mRNA expression of IL1β, IL18 and NLRP3. The underlying rationale for these events is likely explained by a combination of ATP-P2X7R activation, which triggers an influx of Ca^2+^ and K^+^ efflux, combined with caspase 1 mediated cleavage of IL1β and downstream inflammation. Together, increased [Ca^2+^]_i_ and inflammation are each independent triggers of hemichannel opening [66, 67] whilst NFκB mediated NLRP3 priming has been linked to increased Cx43 transcription (NFκB binds to Cx43 promoter) [36]. These observations support the existence of a feedback loop in which sustained NLRP3 activity triggers further NLRP3 priming and activation via increased Cx43 hemichannel mediated ATP release.

To test this hypothesis, ATP-biosensing and carboxyfluorescein dye uptake studies were performed on TGFβ1-treated primary hPTECs pre-incubated with either Peptide5 or specific inhibitors to NLRP3 (CY-09) or caspase 1 (AC-YVAD-CMK). Pharmacological inhibition of NLRP3 and caspase 1 reduced both Cx43 hemichannel mediated dye uptake and ATP release. Furthermore, with NLRP3 activity linked to induction of EMT [64], we observed a Cx43-hemichannel and NLRP3 dependent change in expression of proteins integral to both adherens junction assembly and the ECM. These observations substantiate our findings that disassembly of the adherens (e.g., E-cadherin, β-catenin) and tight (e.g., Zona Occludins) junction complexes are blunted in the heterozygous Cx43^+/−^ UUO model [15]. Despite its role in maintaining cell polarity, disassembly of the adherens junction complex allows for cytosolic translocation of β-catenin, which when activated by Wingless-related integration site (*Wnt*) proteins, promotes the association between NLRP3 and its binding partner ASC, highlighting potential cross talk between each of these events [68]. Furthermore, high levels of extracellular ATP decrease E-cadherin expression and reduce intercellular ligation forces between coupled cells via a P2X7R dependent mechanism [16]. Therefore, Cx43 hemichannel mediated activation of P2X7R, not only contributes to priming and activation of the NLRP3 inflammasome but underlies the reduction in tubule GJIC as previously reported [16, 28].

In summary, for the first time our results provide a definitive link between aberrant Cx43-hemichannel activity and changes that underpin late-stage inflammatory damage in the diseased tubules. We show that aberrant Cx43-hemichannel mediated activity is linked to priming and activation of the NLRP3 inflammasome (Schematic 1), initiating the secretion of inflammatory cytokines and chemo-attractants which activate and recruit F4/80^+^ macrophages and FSP1^+^ fibroblasts in the tubulointerstitium. We report that these effects can be dampened by Cx43-hemichannel blocker Peptide5, and that preventing the release of extracellular ATP, breaks the chronic cycle of inflammatory cytokine release and resultant paracrine mediated signalling between different cell types in and around the tubules. Our findings highlight that Cx43 hemichannels may represent a potential therapeutic target in alleviating sterile inflammation in late-stage CKD and build on observations that have identified a link between Cx43 expression and kidney damage. Whilst our work shows that targeting Cx43 hemichannels prevents the severity of tubulointerstitial fibrosis that develops in UUO mice, future studies are required to determine the efficacy of Peptide5 or alternative Cx43 hemichannel blockers in other models of CKD notably those where full renal function can be assessed.

**Schematic 1:**
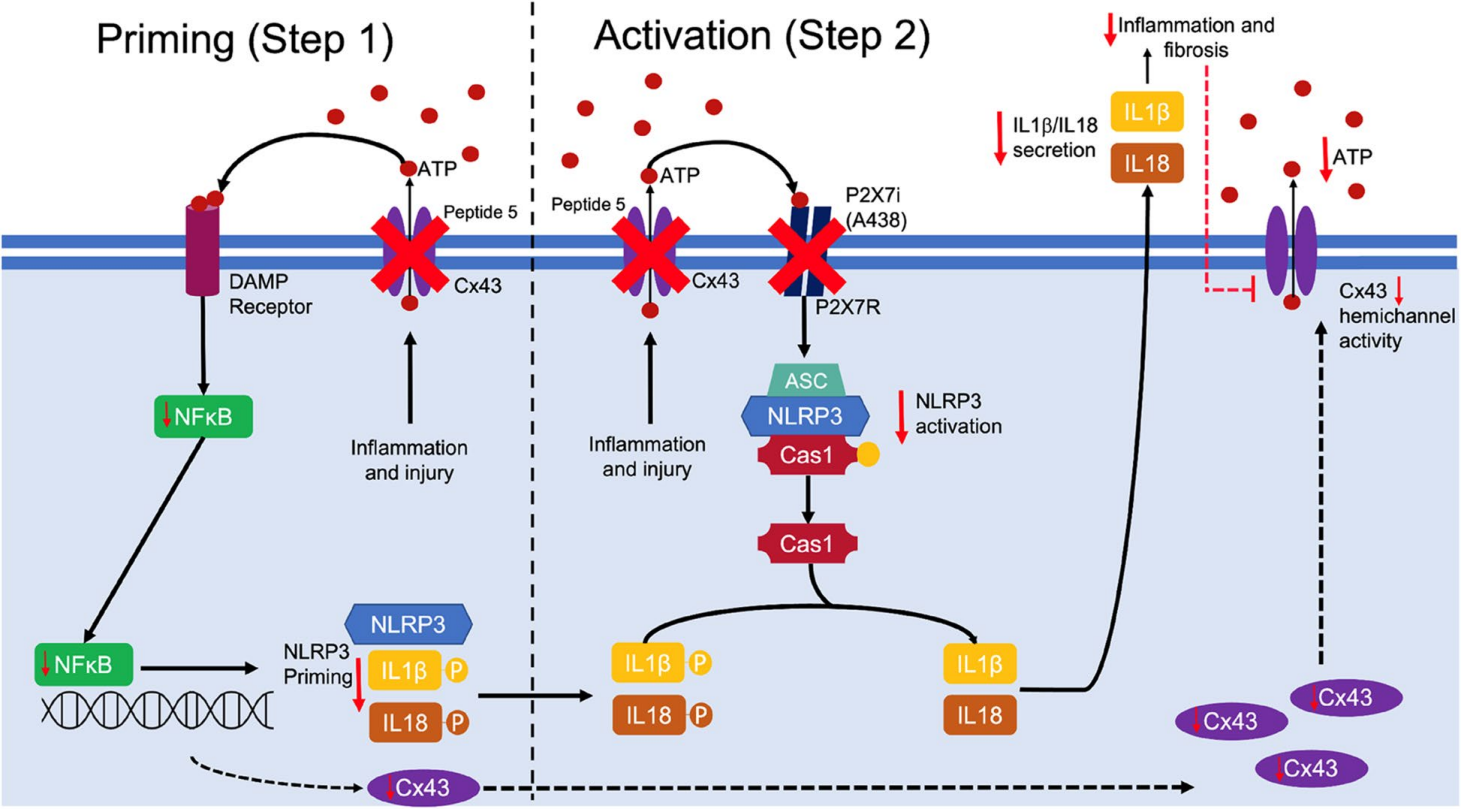
In the diseased kidney tubules, increased Cx43 hemichannel activity is linked to an increase in priming and activation of the NRLP3 inflammasome via NFκB and ATP mediated P2 × 7R activity respectively. This chain of events culminates in caspase 1 mediated cleavage of IL1β, IL18 and activation of downstream inflammatory mediators. In the face of persistent NLRP3 activation, Cx43 hemichannels remain open, perpetuating a viscous cycle of events in which NFκB mediated NLRP3 priming, increases Cx43 expression, with P2X7R mediated NLRP3 activation increasing [Ca^2+^]_i_ and downstream inflammation. Peptide5 successfully blocks these ATP-driven events, decreasing NLRP3 priming and activation. These observations are paralleled by a reduction in tubule inflammation and paracrine mediated signaling, as evidenced by a reduction in the secretion of soluble signaling mediators, decreased fibroblast activation and reduced macrophage number

### Supplementary Information

**Additional file 1: Figure 1.** Generation of a transgenic (Pax8-rtTA-cre:cx43 flox Cx43^-/-^) mouse model. (Panel a) Scheme of the transgenic construct used to delete Cx43 in renal tubular cells. The reverse tetracycline-dependent transactivator (rtTA) drives the expression of Cre recombinase specifically in the renal tubular compartment (controlled by Pax8 promoter) after doxycycline administration. (Panel b) An illustration of the experimental design using WT controls and mice with and without Cx43 tubule-specific deletion, subjected to UUO. Kidneys were collected after 10 days of UUO for RNA extraction, renal morphometry and immunostainings. (Panel c) Connexin 43 immunostainings (in green) were performed on cryosections from injured kidneys of mice with and without Cx43 tubule-specific deletion after 10 days of UUO. Connexin 43 is undetectable in UUO-Cx43^-/-^ mice. Sections were counterstained with Evans Blue (red). (Panel d) Connexin 43 mRNA increased expression was blunted in injured kidneys of mice with Cx43 tubule-specific deletion after 10 days of UUO.

**Additional file 2: Figure 2.** PYCARD expression is increased in biopsy material from patients with CKD. Analysis of a published transcriptomic dataset shows that the NLRP3 binding partner ASC, alternatively called PYCARD, exhibits increased expression in kidneys of individuals with CKD (*n*=48 patients) as compared to healthy controls (*n*=5). Statistical analysis was performed using an unpaired t-test with Welch’s correction. *P<0.05, **P<0.01, ***P<0.005, and ****P<0.001.

**Additional file 3: Figure 3.** Peptide 5 protocol. (Panel a) Experimental design for the Cx43 hemichannel specific blocker Peptide5 (P5). Peptide5 was administered intraperitoneally twice a day for 9 days following UUO. All mice were sacrificed at day 10. (Panel b) Kidneys from wild type (WT) controls and mice with or without Peptide5 injections were collected after 10 days of UUO for RNA extraction, renal morphometry and immunostainings.

## Data Availability

The datasets used and/or analysed during the current study are available from the corresponding author on reasonable request.

## References

[1] Speer T, Dimmeler S, Schunk SJ, Fliser D, Ridker PM (2022). Targeting innate immunity-driven inflammation in CKD and cardiovascular disease. Nat Rev Nephrol.

[2] Mylonas KJ, O’Sullivan ED, Humphries D, Baird DP, Docherty MH, Neely SA (2021). Cellular senescence inhibits renal regeneration after injury in mice, with senolytic treatment promoting repair. Sci Transl Med.

[3] Vilaysane A, Chun J, Seamone ME, Wang W, Chin R, Hirota S (2010). The NLRP3 inflammasome promotes renal inflammation and contributes to CKD. J Am Soc Nephrol.

[4] Olsen MB, Gregersen I, Sandanger Ø, Yang K, Sokolova M, Halvorsen BE (2021). Targeting the inflammasome in cardiovascular disease. JACC Basic Transl Sci.

[5] Bugyei-Twum A, Abadeh A, Thai K, Zhang Y, Mitchell M, Kabir G (2016). Suppression of NLRP3 inflammasome activation ameliorates chronic kidney disease-induced cardiac fibrosis and diastolic dysfunction. Sci Rep.

[6] Zeisberg M, Neilson EG (2010). Mechanisms of tubulointerstitial fibrosis. J Am Soc Nephrol.

[7] Luo C, Zhou S, Zhou Z, Liu Y, Yang L, Liu J (2018). Wnt9a promotes renal fibrosis by accelerating cellular senescence in tubular epithelial cells. J Am Soc Nephrol.

[8] Chen XJ, Kim SR, Jiang K, Ferguson CM, Tang H, Zhu XY (2021). Renovascular disease induces senescence in renal scattered tubular-like cells and impairs their reparative potency. Hypertension.

[9] Kim SR, Jiang K, Ogrodnik M, Chen X, Zhu XY, Lohmeier H (2019). Increased renal cellular senescence in murine high-fat diet: effect of the senolytic drug quercetin. Transl Res.

[10] Ridker PM, MacFadyen JG, Glynn RJ, Koenig W, Libby P, Everett BM (2018). Inhibition of interleukin-1β by Canakinumab and cardiovascular outcomes in patients with chronic kidney disease. J Am Coll Cardiol.

[11] Guo A, Zhang H, Li H, Chiu A, García-Rodríguez C, Lagos CF (2022). Inhibition of connexin hemichannels alleviates neuroinflammation and hyperexcitability in temporal lobe epilepsy. Proc Natl Acad Sci USA.

[12] Varela-Eirín M, Carpintero-Fernández P, Guitián-Caamaño A, Varela-Vázquez A, García-Yuste A, Sánchez-Temprano A (2022). Extracellular vesicles enriched in connexin 43 promote a senescent phenotype in bone and synovial cells contributing to osteoarthritis progression. Cell Death Dis.

[13] Bhattacharyya A, Torre P, Yadav P, Boostanpour K, Chen TY, Tsukui T (2022). Macrophage Cx43 is necessary for fibroblast cytosolic calcium and lung fibrosis after injury. Front Immunol.

[14] Abed A, Toubas J, Kavvadas P, Authier F, Cathelin D, Alfieri C (2014). Targeting connexin 43 protects against the progression of experimental chronic kidney disease in mice. Kidney Int.

[15] Price GW, Chadjichristos CE, Kavvadas P, Tang SCW, Yiu WH, Green CR (2020). Blocking Connexin-43 mediated hemichannel activity protects against early tubular injury in experimental chronic kidney disease. Cell Commun Signal.

[16] Siamantouras E, Price GW, Potter JA, Hills CE, Squires PE (2019). Purinergic receptor (P2 × 7) activation reduces cell-cell adhesion between tubular epithelial cells of the proximal kidney. Nanomedicine: Nanatechnol Biology Med.

[17] Xu H, Wang M, Li Y, Shi M, Wang Z, Cao C (2022). Blocking connexin 43 and its promotion of ATP release from renal tubular epithelial cells ameliorates renal fibrosis. Cell Death Dis.

[18] Kuo CY, Maran JJ, Jamieson EG, Rupenthal ID, Murphy R, Mugisho OO (2022). Characterization of NLRP3 inflammasome activation in the onset of diabetic retinopathy. Int J Mol Sci.

[19] Mat Nor MN, Rupenthal ID, Green CR, Acosta ML (2020). Connexin hemichannel block using orally delivered Tonabersat improves outcomes in animal models of retinal disease. Neurotherapeutics.

[20] Rodríguez-Poncelas A, Mundet-Tudurí X, Miravet-Jiménez S, Casellas A, Barrot-De la Puente JF, Franch-Nadal J (2016). Chronic kidney Disease and Diabetic Retinopathy in patients with type 2 diabetes. PLoS ONE.

[21] O’Carroll SJ, Alkadhi M, Nicholson LF, Green CR (2008). Connexin 43 mimetic peptides reduce swelling, astrogliosis, and neuronal cell death after spinal cord injury. Cell Commun Adhes.

[22] Laird DW, Lampe PD (2018). Therapeutic strategies targeting connexins. Nat Rev Drug Discov.

[23] Kormann R, Kavvadas P, Placier S, Vandermeersch S, Dorison A, Dussaule JC (2020). Periostin promotes cell proliferation and macrophage polarization to drive repair after AKI. J Am Soc Nephrol.

[24] Kavvadas P, Abed A, Poulain C, Authier F, Labéjof LP, Calmont A (2017). Decreased expression of Connexin 43 blunts the progression of experimental GN. J Am Soc Nephrol.

[25] Nakagawa S, Nishihara K, Miyata H, Shinke H, Tomita E, Kajiwara M (2015). Molecular markers of tubulointerstitial fibrosis and tubular cell damage in patients with chronic kidney disease. PLoS ONE.

[26] Schmid H, Boucherot A, Yasuda Y, Henger A, Brunner B, Eichinger F (2006). Modular activation of nuclear factor-κB transcriptional programs in human diabetic nephropathy. Diabetes.

[27] Ju W, Nair V, Smith S, Zhu L, Shedden K, Song PXK (2015). ERCB, C-PROBE, NEPTUNE, and PKU-IgAN Consortium. Tissue transcriptome-driven identification of epidermal growth factor as a chronic kidney disease biomarker. Sci Transl Med.

[28] Hills CE, Siamantouras E, Smith SW, Cockwell P, Liu KK, Squires PE (2012). TGFβ modulates cell-to-cell communication in early epithelial-to-mesenchymal transition. Diabetologia.

[29] Price GW, Potter JA, Williams BM, Cliff CL, Wall MJ, Hills CE (2021). Examining local cell-to-cell signalling in the kidney using ATP biosensing. Methods Mol Biol.

[30] Potter JA, Price GW, Cliff CL, Williams BM, Hills CE, Squires PE (2021). Carboxyfluorescein dye uptake to measure connexin-mediated hemichannel activity in cultured cells. Bio Protoc.

[31] Anders HJ, Suarez-Alvarez B, Grigorescu M, Foresto-Neto O, Steiger S, Desai J (2018). The macrophage phenotype and inflammasome component NLRP3 contributes to nephrocalcinosis-related chronic kidney disease independent from IL-1-mediated tissue injury. Kidney Int.

[32] Nor NM, Guo CX, Rupenthal ID, Chen YS, Green CR, Acosta ML (2018). Sustained connexin43 mimetic peptide release from loaded nanoparticles reduces retinal and choroidal photodamage. Invest Ophthalmol Vis Sci.

[33] Squires PE, Price GW, Mouritzen U, Potter JA, Williams BM, Hills CE (2021). Danegaptide prevents TGFβ1-induced damage in human proximal tubule epithelial cells of the kidney. Int J Mol Sci.

[34] Mugisho OO, Green CR, Kho DT, Zhang J, Graham ES, Acosta ML (2018). The inflammasome pathway is amplified and perpetuated in an autocrine manner through connexin43 hemichannel mediated ATP release. Biochim Biophys Acta Gen Subj.

[35] Kong H, Zhao H, Chen T, Song Y, Cui Y (2022). Targeted P2 × 7/NLRP3 signaling pathway against inflammation, apoptosis, and pyroptosis of retinal endothelial cells in diabetic retinopathy. Cell Death Dis.

[36] Alonso F, Krattinger N, Mazzolai L, Simon A, Waeber G, Meda P (2010). An angiotensin II- and NF-kappaB-dependent mechanism increases connexin 43 in murine arteries targeted by renin-dependent hypertension. Cardiovasc Res.

[37] Sáez JC, Contreras-Duarte S, Labra VC, Santibañez CA, Mellado LA, Inostroza CA (2020). Interferon-γ and high glucose-induced opening of Cx43 hemichannels causes endothelial cell dysfunction and damage. Biochim Biophys Acta Mol Cell Res.

[38] Sáez JC, Contreras-Duarte S, Gómez GI, Labra VC, Santibañez CA, Gajardo-Gómez R (2018). Connexin 43 hemichannel activity promoted by pro-inflammatory cytokines and high glucose alters endothelial cell function. Front Immunol.

[39] Tesch GH (2008). MCP-1/CCL2: a new diagnostic marker and therapeutic target for progressive renal injury in diabetic nephropathy. Am J Physiol Renal Physiol.

[40] Huen SC, Huynh L, Marlier A, Lee Y, Moeckel GW, Cantley LG (2015). GM-CSF promotes macrophage alternative activation after renal ischemia/reperfusion injury. J Am Soc Nephrol.

[41] Ye T, Zhang J, Wu D, Shi J, Kuang Z, Ma Y (2022). Empagliflozin attenuates obesity-related kidney dysfunction and NLRP3 inflammasome activity through the HO-1-adiponectin axis. Front Endocrinol (Lausanne).

[42] Taguchi S, Azushima K, Yamaji T, Urate S, Suzuki T, Abe E (2021). Effects of tumor necrosis factor-α inhibition on kidney fibrosis and inflammation in a mouse model of aristolochic acid nephropathy. Sci Rep.

[43] Schütte-Nütgen K, Edeling M, Kentrup D, Heitplatz B, Van Marck V, Zarbock A (2022). Interleukin 24 promotes cell death in renal epithelial cells and is associated with acute renal injury. Am J Transplant.

[44] Xu S, Yang X, Chen Q, Liu Z, Chen Y, Yao X (2022). Leukemia inhibitory factor is a therapeutic target for renal interstitial fibrosis. EBioMedicine.

[45] Axelsson J, Bergsten A, Qureshi AR, Heimbürger O, Bárány P, Lönnqvist F (2006). Elevated resistin levels in chronic kidney disease are associated with decreased glomerular filtration rate and inflammation, but not with insulin resistance. Kidney Int.

[46] Disteldorf EM, Krebs CF, Paust HJ, Turner JE, Nouailles G, Tittel A (2015). CXCL5 drives neutrophil recruitment in TH17-mediated GN. J Am Soc Nephrol.

[47] Varela-Eirín M, Carpintero-Fernández P, Sánchez-Temprano A, Varela-Vázquez A, Paíno CL, Casado-Díaz A (2020). Senolytic activity of small molecular polyphenols from olive restores chondrocyte redifferentiation and promotes a pro-regenerative environment in osteoarthritis. Aging.

[48] Martínez-Klimova E, Aparicio-Trejo OE, Tapia E, Pedraza-Chaverri J (2019). Unilateral ureteral obstruction as a model to investigate fibrosis-attenuating treatments. Biomolecules.

[49] Kim Y, Griffin JM, Harris PW, Chan SH, Nicholson LF, Brimble MA (2017). Characterizing the mode of action of extracellular Connexin43 channel blocking mimetic peptides in an in vitro ischemia injury model. Biochim Biophys Acta Gen Subj.

[50] Williams BM, Cliff CL, Demirel I, Squires PE, Hills CE (2022). Blocking connexin 43 hemichannel-mediated ATP release reduces communication within and between tubular epithelial cells and medullary fibroblasts in a model of diabetic nephropathy. Diabet Med.

[51] Chung AC, Lan HY (2011). Chemokines in renal injury. J Am Soc Nephrol.

[52] Li HD, You YK, Shao BY, Wu WF, Wang YF, Guo JB (2022). Roles and cross talks of macrophages in diabetic nephropathy. Front Immunol.

[53] Sakai N, Tager AM (2013). Fibrosis of two: epithelial cell-fibroblast interactions in pulmonary fibrosis. Biochem Biophys Acta.

[54] Bednarczyk RB, Tuli NY, Hanly EK, Rahoma GB, Maniyar R, Mittelman A (2018). Macrophage inflammatory factors promote epithelial-mesenchymal transition in breast cancer. Oncotarget.

[55] Wang YY, Jiang H, Pan J, Huang XR, Wang YC, Huang HF (2017). Macrophage-to-myofibroblast transition contributes to interstitial fibrosis in chronic renal allograft injury. J Am Soc Nephrol.

[56] Mugisho OO, Green CR (2022). The NLRP3 inflammasome in age-related eye disease: evidence-based connexin hemichannel therapeutics. Exp Eye Res.

[57] Mugisho OO, Aryal J, Shorne A, Lyon H, Acosta ML, Green CR (2023). Orally delivered connexin43 hemichannel blocker, Tonabersat, inhibits vascular breakdown and inflammasome activation in a mouse model of diabetic retinopathy. Int J Mol Sci.

[58] Ju H, Wang Y, Shi Q, Zhou Y, Ma R, Wu P (2019). Inhibition of connexin 43 hemichannels improves postoperative cognitive function in aged mice. Am J Transl Res.

[59] Ridker PM, Tuttle KR, Perkovic V, Libby P, MacFadyen JG (2022). Inflammation drives residual risk in chronic kidney disease: a CANTOS substudy. Eur Heart J.

[60] Ridker PM, Devalaraja M, Baeres FMM, Engelmann MDM, Hovingh GK, Ivkovic M (2021). IL-6 inhibition with Ziltivekimab in patients at high atherosclerotic risk (RESCUE): a double-blind, randomised, placebo-controlled, phase 2 trial. Lancet.

[61] Qian Y, Qian C, Xie K, Fan Q, Yan Y, Lu R (2021). P2 × 7 receptor signaling promotes inflammation in renal parenchymal cells suffering from ischemia-reperfusion injury. Cell Death Dis.

[62] Romero A, Dongil P, Valencia I, Vallejo S, Hipólito-Luengo ÁS, Díaz-Araya G (2022). Pharmacological blockade of NLRP3 inflammasome/IL-1β-positive loop mitigates endothelial cell senescence and dysfunction. Aging Dis.

[63] Acosta JC, Banito A, Wuestefeld T, Georgilis A, Janich P, Morton JP (2013). A complex secretory program orchestrated by the inflammasome controls paracrine senescence. Nat Cell Biol.

[64] Alyaseer AAA, de Lima MHS, Braga TT (2020). The role of NLRP3 inflammasome activation in the epithelial to mesenchymal transition process during the fibrosis. Front Immunol.

[65] Menzies RI, Booth JWR, Mullins JJ, Bailey MA, Tam FWK, Norman JT (2017). Hyperglycemia-induced renal P2 × 7 receptor activation enhances diabetes-related injury. EBioMedicine.

[66] Lagos-Cabré R, Brenet M, Díaz J, Pérez RD, Pérez LA, Herrera-Molina R (2018). Intracellular Ca^2+^ increases and connexin 43 hemichannel opening are necessary but not sufficient for Thy-1-induced astrocyte migration. Int J Mol Sci.

[67] Van Campenhout R, Gomes AR, De Groof TWM, Muyldermans S, Devoogdt N, Vinken M (2021). Mechanisms underlying connexin hemichannel activation in disease. Int J Mol Sci.

[68] Huang L, Luo R, Li J, Wang D, Zhang Y, Liu L (2020). β-catenin promotes NLRP3 inflammasome activation via increasing the association between NLRP3 and ASC. Mol Immunol.

