# Supplementary figures and images for: Connexin-43 hemichannels orchestrate NOD-like receptor protein-3 (NLRP3) inflammasome activation and sterile inflammation in tubular injury
Source: Cell Commun Signal. 2023 Sep 28;21:263. doi: 10.1186/s12964-023-01245-7 (PMC10536814; doi:10.1186/s12964-023-01245-7)

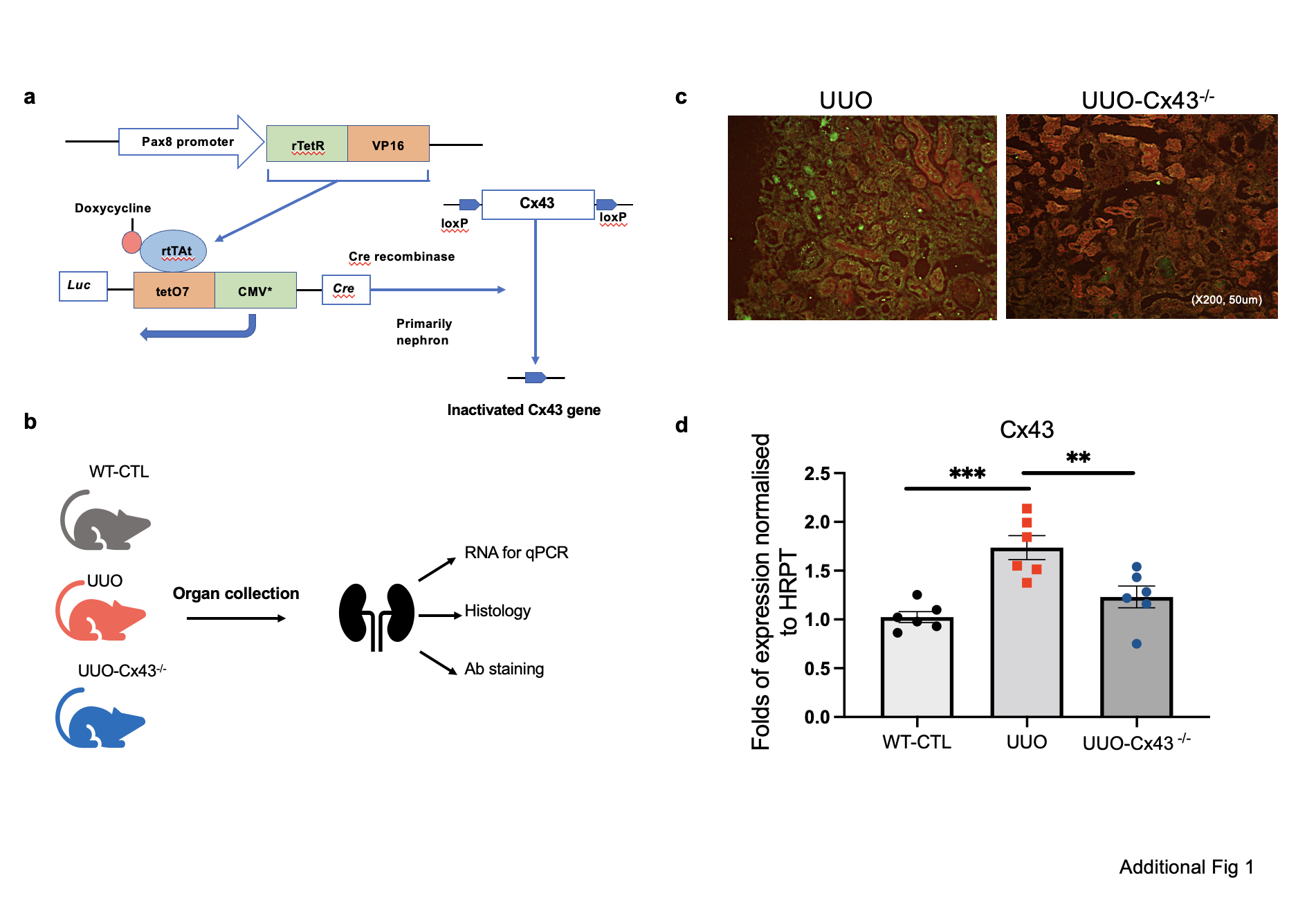

Supplement: Supplementary file 2 — Additional file 1: Figure 1. Generation of a transgenic (Pax8-rtTA-cre:cx43 flox Cx43-/-) mouse model. (Panel a) Scheme of the transgenic construct used to delete Cx43 in renal tubular cells. The reverse tetracycline-dependent transactivator (rtTA) drives the expression of Cre recombinase specifically in the renal tubular compartment (controlled by Pax8 promoter) after doxycycline administration. (Panel b) An illustration of the experimental design using WT controls and mice with and without Cx43 tubule-specific deletion, subjected to UUO. Kidneys were collected after 10 days of UUO for RNA extraction, renal morphometry and immunostainings. (Panel c) Connexin 43 immunostainings (in green) were performed on cryosections from injured kidneys of mice with and without Cx43 tubule-specific deletion after 10 days of UUO. Connexin 43 is undetectable in UUO-Cx43-/- mice. Sections were counterstained with Evans Blue (red). (Panel d) Connexin 43 mRNA increased expression was blunted in injured kidneys of mice with Cx43 tubule-specific deletion after 10 days of UUO. [file 12964_2023_1245_MOESM1_ESM.tiff]

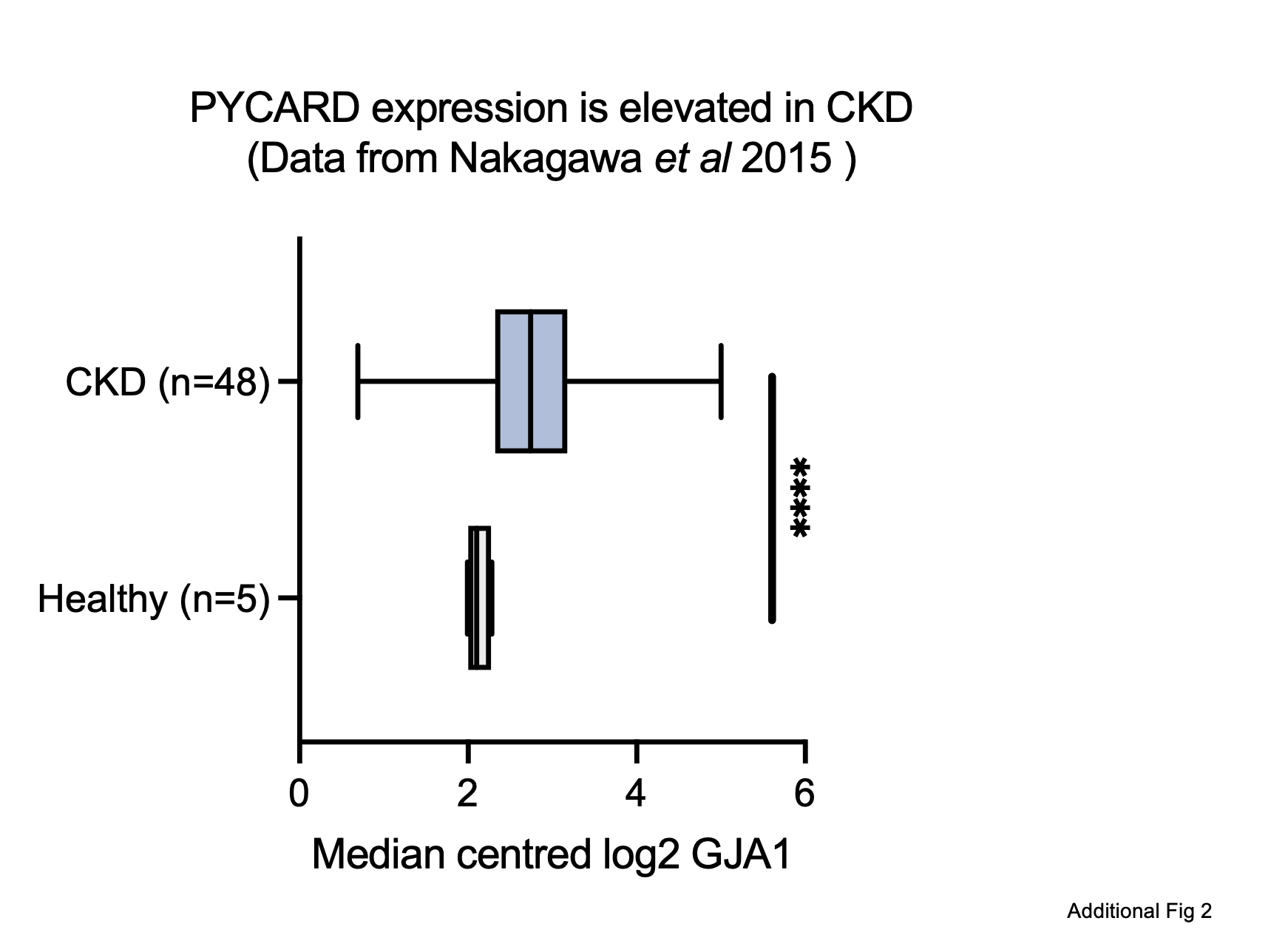

Supplement: Supplementary file 3 — Additional file 2: Figure 2. PYCARD expression is increased in biopsy material from patients with CKD. Analysis of a published transcriptomic dataset shows that the NLRP3 binding partner ASC, alternatively called PYCARD, exhibits increased expression in kidneys of individuals with CKD (n=48 patients) as compared to healthy controls (n=5). Statistical analysis was performed using an unpaired t-test with Welch’s correction. *P<0.05, **P<0.01, ***P<0.005, and ****P<0.001. [file 12964_2023_1245_MOESM2_ESM.tiff]

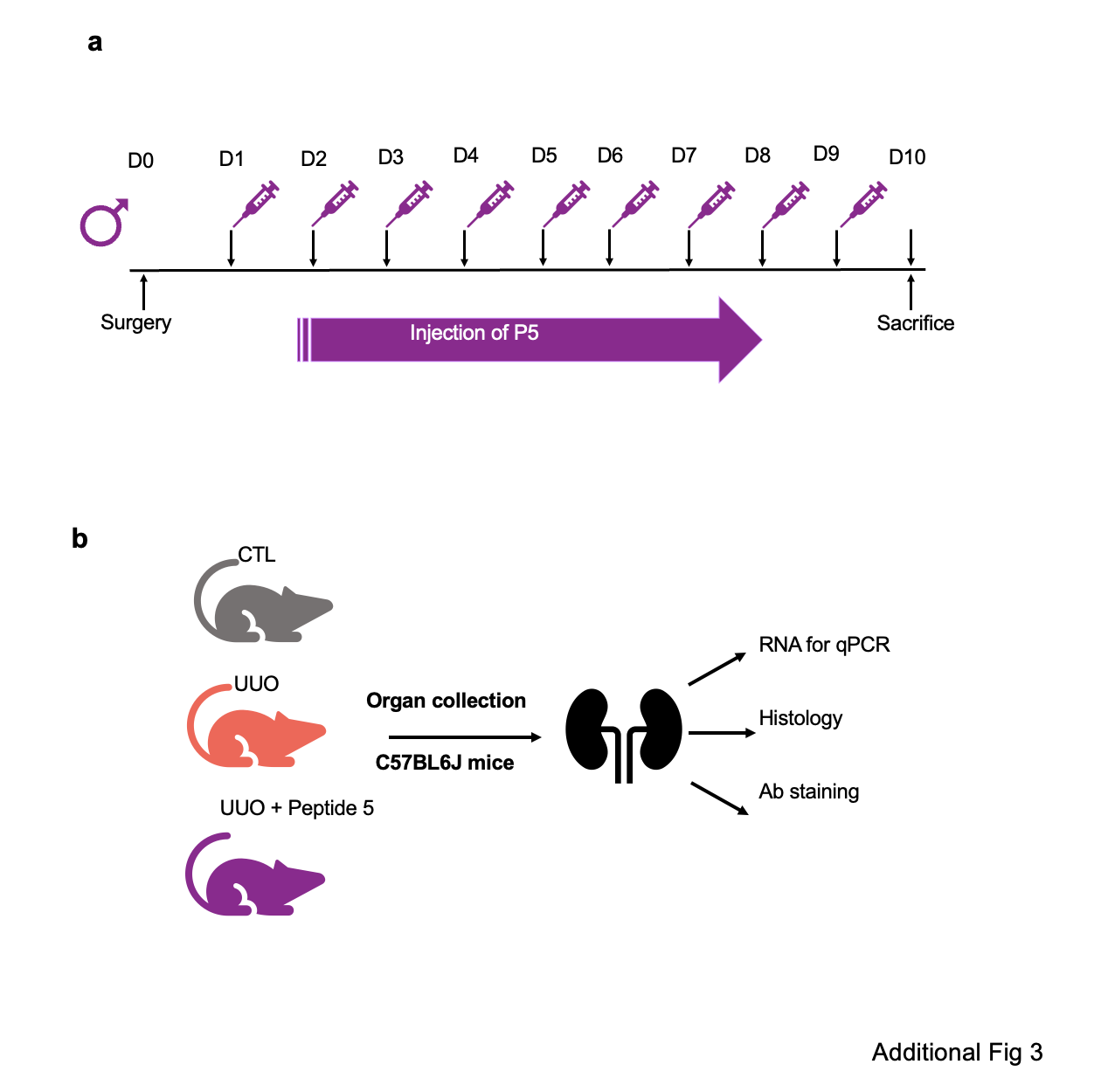

Supplement: Supplementary file 4 — Additional file 3: Figure 3. Peptide 5 protocol. (Panel a) Experimental design for the Cx43 hemichannel specific blocker Peptide5 (P5). Peptide5 was administered intraperitoneally twice a day for 9 days following UUO. All mice were sacrificed at day 10. (Panel b) Kidneys from wild type (WT) controls and mice with or without Peptide5 injections were collected after 10 days of UUO for RNA extraction, renal morphometry and immunostainings. [file 12964_2023_1245_MOESM3_ESM.tiff]
